# Trifluridine/tipiracil induces ferroptosis by targeting p53 via the p53-SLC7A11 axis in colorectal cancer 3D organoids

**DOI:** 10.1038/s41419-025-07541-z

**Published:** 2025-04-05

**Authors:** Maosen Huang, Yancen Wu, Xiaoxia Wei, Linyao Cheng, Lihua Fu, Haochao Yan, Wene Wei, Bo Li, Haiming Ru, Xianwei Mo, Weizhong Tang, Zijie Su, Linhai Yan

**Affiliations:** 1 Department of Gastrointestinal Surgery, Guangxi Medical University Cancer Hospital, Nanning, 530021 Guangxi Zhuang Autonomous Region China; 2 Guangxi Clinical Research Center for Colorectal Cancer, Nanning, 530021 Guangxi Zhuang Autonomous Region China; 3 Department of Experimental Research, Guangxi Medical University Cancer Hospital, Nanning, 530021 Guangxi Zhuang Autonomous Region China; 4 Liaoning Provincial Engineering Laboratory of Anti-tumor Immunity and Molecular Theranostics Technology, Collaborative Innovation Center for Age-related Disease, Life Science Institute of Jinzhou Medical University, Jinzhou, 121001 Liaoning China

**Keywords:** Experimental models of disease, Chemotherapy, Rectal cancer

## Abstract

Trifluridine/Tipiracil (FTD/TPI, TAS102) has been approved for the treatment of patients with colorectal cancer (CRC) for its promising anticancer activity enabled by its incorporation into double strands during DNA synthesis. However, the mechanisms underlying the anticancer targets of FTD/TPI remain not fully understood. Here we report our observation of the activation of ferroptosis in CRC by FTD/TPI. Mechanistically, FTD/TPI directly promotes the ubiquitination and degradation of MDM2, thereby stabilizing the p53. Nuclear accumulation of p53 subsequently downregulates SLC7A11 expression, leading to ferroptosis. Furthermore, we observed that FTD/TPI combined with sulfasalazine (SAS), a system Xc^–^ inhibitor, works in a synergistic manner to induce ferroptosis and further inhibit the proliferation of CRC cells. Finally, we confirmed the synergistic effect of SAS and FTD/TPI on patient-derived organoids in vitro and patient-derived xenograft mouse models in vivo. Our findings are the first to reveal that FTD/TPI induces ferroptosis via the p53-SLC7A11 axis and that SAS enhances the sensitivity and therapeutic effect of FTD/TPI. These findings suggest that the synergistic effect of FTD/TPI and SAS may represent a new therapeutic strategy for patients with CRC.

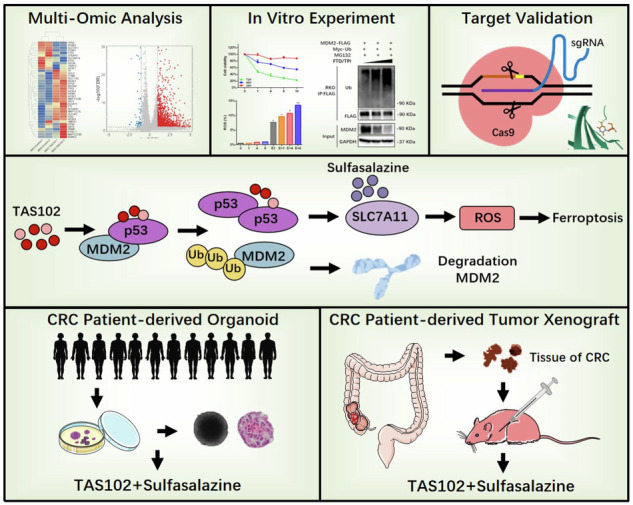

## Introduction

The incidence of colorectal cancer (CRC) is increasing annually. CRC is now the third most common type of cancer worldwide and the second most common cause of cancer-related death, accounting for ~10% of all cancer cases [1, 2]. Approximately 25% of patients with primary CRC eventually develop metastatic disease. After the failure of multi-line chemotherapy, these patients face a poor prognosis of less than 20%, termed refractory CRC [3]. First-line drugs or tyrosine kinase inhibitors (TKIs) are typically used to treat refractory CRC. However, more than 80% of patients with advanced refractory CRC have no usable targets, so they cannot receive precise treatment. Moreover, repeated use of fluorouracil-based drugs poses a major clinical challenge due to intrinsic and acquired resistance [4, 5]. Furthermore, the indiscriminate combination of multiple chemotherapeutic agents and targeted therapies leads to a higher incidence of adverse drug reactions. Therefore, there is an urgent need for the development of new and effective strategies and approaches to treat patients with advanced CRC.

Trifluridine/Tipiracil (FTD/TPI, TAS102) is an oral combination formulation of trifluridine (FTD) and tipiracil (TPI) in a molar ratio of 1:0.5 that has been approved for patients with advanced metastatic CRC. Recent (2023) clinical trials (ASCO and SUNLIGHT) have shown promising prospects for its use in the treatment of cancer [6]. In contrast to conventional traditional cytotoxic drugs, FTD/TPI is a novel oral cytotoxic agent with a unique anti-tumor mechanism. FTD is activated by thymidine kinase (TK) and forms trifluridine monophosphate (F3dTMP), which inhibits thymidylate synthase (TS), resulting in impaired DNA synthesis [7]. The addition of TPI can improve the bioavailability of FTD. Although 5-fluorouracil (5-FU) also inhibits TS synthesis and thus induces cell apoptosis, its mechanism of action involves intracellular conversion to fluorodeoxyuridine monophosphate (FdUMP). FTD has been confirmed to be incorporated into DNA to a much greater extent, and the anti-tumor effect of FTD is not exclusively due to the induction of DNA strand breaks [8]. In addition, low doses of FTD can be incorporated into cellular DNA, thereby exerting direct anti-tumor activity and a sustained effect [9]. However, the specific mechanisms of FTD/TPI action in CRC are poorly understood.

Ferroptosis was first proposed in 2012 by Dixon et al., who described it as a form of regulated cell death characterized by metabolic dysfunction of intracellular lipid peroxides. This dysfunction leads to excessive production of lipids under the catalysis of iron ions, disrupting the balance between the regulation of oxidative damage and antioxidant defense, ultimately leading to cell death [10]. Pharmacological regulation of iron deficiency anemia by induction or inhibition holds great potential for treating tumors and overcoming tumor resistance [11–13]. Studies have demonstrated that the combined use of experimental ferroptosis reagents, such as erastin and RSL3; clinically approved drugs, such as sorafenib and sulfasalazine (SAS); and ionizing radiation can inhibit tumor growth [10, 14, 15]. Several metabolic pathways are involved in the development of ferroptosis, including GSH/GPX4 regulation, iron metabolism, and lipid metabolism. Metabolic dysfunction, gene mutations, and imbalances in ferroptosis defense are considered sensitive factors that influence the susceptibility of CRC to ferroptosis [16–18].

Ferroptosis can induce CRC cell death and reverse drug resistance. Research has shown that activation of NRF2 and p53, which inhibit the xCT/GPX4 system and promote the accumulation of reactive oxygen species (ROS), sensitizes CRC cells to ferroptosis activators [19–22]. CRC growth is associated with iron imbalance, and excessive iron levels can generate ROS through the Fenton reaction, ultimately leading to cell sensitivity to chemotherapeutic agents [23–27]. In vitro suppression of ferroptosis can trigger the production of 5-FU and oxaliplatin resistance mechanisms in CRC, which is attributed to decreased sensitivity to drugs. This could be due to inhibition of nuclear input of NRF2 and downregulation of GPX4 [28–30]. Given the presence of multiple drug-resistant mechanisms in patients with advanced CRC and the lack of precise therapeutic targets, we aimed to reverse CRC resistance using ferroptosis activators to sensitize cancer cells to chemotherapy and targeted treatments.

In this study, we found that FTD/TPI inhibits tumor cell growth by activating ferroptosis via the p53-SLC7A11 axis. We hypothesized that FTD/TPI could serve as an alternative therapeutic approach targeting the p53/MDM2 pathway. We observed that SAS synergistically enhanced the ferroptosis effect of FTD/TPI, thereby improving its cytotoxic efficacy against CRC cells. This combined therapy offers new clinical strategies and treatment options for patients with advanced CRC and chemoresistance.

## Materials and methods

### Cell lines

The human CRC cell lines RKO and HT29 and the human embryonic kidney cell line HEK293T were cultured in DMEM medium (Gibco), HCT116 and DLD1 cells in RPMI-1640 medium (Gibco). Cells were supplemented with 10% fetal bovine serum (Gibco) and 10,000 U/mL Penicillin-Streptomycin (Gibco), and incubated at 37 °C with 5% CO_2_. All cells were obtained from the American Type Culture Collection (ATCC, Manassas, VA, USA) and tested for mycoplasma.

### Chemical reagents, antibodies, and plasmids

TAS102 (FTD/TPI), MG132, cycloheximide, and Bafilomycin A1 were obtained from Selleck ((Houston, TX, USA). Ferrostatin-1, Necrosulfonamide, and Z-VAD-FMK were purchased from Targetmol (Wellesley Hills, MA, USA). Erastin, sulfasalazine, artemisinin, and dihydroartemisinin were obtained from MedChemExpress (Monmouth Junction, NJ, USA). The following primary antibodies were used: p53 (Santa Cruz, sc-126), ACSL4 (Santa Cruz, sc-365230), MDM2 (Cell Signaling Technology, #86934), SLC7A11 (Cell Signaling Technology, #12691), Ubiquitin (Proteintech, 10201- 2-AP), GPX4 (Abcam, ab125066), GAPDH (Proteintech, 60004-1-Ig), α-Tubulin (Proteintech, 66031-1-Ig). The plasmid of human MDM2 constructed in pCMV-MCS-3×Flag vector was a gift from Mailgene (Mailgene, Beijing, China, MH03043).

### Cellular thermal shift assay (CETSA)

In order to determine the targeting effect of FTD/TPI on p53, a cellular thermal shift assay was performed as described [31, 32]. RKO and HCT116 cells were placed in a 15 cm Petri dish and treated with a specific concentration of FTD/TPI or DMSO for 1 h. Cells were collected and washed with PBS once, and then suspended in PBS with protease inhibitors (Monmouth Junction, NJ, USA), maintaining the same dose of FTD/TPI or DMSO as the initial treatment. The cell suspension was distributed in five 1.5 mL PCR tubes at different designated temperatures. The samples were heated for 2 min in a metal bath heater at different specified temperatures. Then, the tubes were removed and immediately incubated at room temperature for 3 min. Three repeated freeze-thaw lysis of cells in liquid nitrogen were performed. The cell lysate was collected by centrifuging at 20,000×*g* for 20 min at 4 °C. The sample of cell lysate was boiled in a loading buffer at 95 °C for 5 min for western blotting analysis, and the dissolution curve of the p53 protein was prepared.

### Molecular docking

To evaluate the binding affinity and interaction mode between the candidate TAS102 and its target p53, we used Autodock Vina 1.2.2, a computer software for protein-ligand docking. The molecular structure of FTD (PubChem CID: 6256) was obtained from the PubChem Compound database. The 3D coordinates of the protein p53 (PDB: 8DC8) were downloaded from the Protein Data Bank. The protein and ligand files were prepared by converting all protein and molecule files to the PDBQT format, removing all water molecules, and adding polar hydrogen atoms. The grid box was centered to cover the structural domain of each protein and accommodate free molecular motion. The docking pocket was set as a square pocket with dimensions of 30 Å × 30 Å × 30 Å and a grid spacing of 0.05 nm. Molecular docking studies were performed using Autodock Vina 1.2.2 for model visualization.

### Transmission electron microscopy

RKO and HCT116 cells were placed in a 6 cm Petri dish and treated with a specific concentration of FTD/TPI or DMSO. Cells were pre-embedded with agar, fixed, and dehydrated at room temperature: The sample was infiltrated and embedded, polymerized, and ultrathin sectioned for staining. The copper mesh was placed in a 2% uranium acetate saturated alcohol solution and a 2.6% lead citrate solution to avoid carbon dioxide and light for 8 min, respectively. Clean and dry overnight at room temperature. Photographs were captured with transmission electron microscopy (Hitachi, HT7800) under 2500, 7000, and 15,000 folds of a microscope. The mitochondrial volume and mitochondrial membrane density of the cells were managed with Image J software.

### Quantitative real-time PCR

Total RNA was isolated from cells using TRIzol reagent (Invitrogen), and cDNA was generated using a reverse transcription kit (TaKaRa). Real-time fluorescence quantitative PCR was performed with primers (Supplementary Table S1) using SYBR Premix Ex TaqII (Promega), and three duplicate samples were run on a software 7500 instrument. Normalize the threshold period (Ct) of the target gene to the threshold of GAPDH and use 2^−ΔΔ Ct^ to calculate the relative expression level of target genes.

### Western blotting

Use RIPA buffer to lyse proteins in cells, which contains 50 mM Tris HCl, pH 7.4, 150 mM NaCl, 1% Triton X-100, 1% Na deoxycholate, 1 mM EDTA, 0.1% SDS, and add protease inhibitors to obtain proteins through ultrasonic lysis. The protein was then loaded in equal amounts and separated with polyacrylamide gel. Then transfer the protein to the PVDF membrane. After incubating the first and second antibodies, they reacted with the ECL solution and detected signals in the Biorad chemidoc MP system.

### CRISPR knock out cell line

TP53 was knocked out in DLD1 cells by using CRISPR/Cas9 technology. The single-guide RNA targeting sequence GATCCACTCACAGTTTCCAT was cloned into the LentiCRISPRv2 vector to obtain the TP53 KO plasmid. For lentiviral production, HEK293T cells were transfected with a packaging plasmid (psPAX2), an envelope plasmid (pMD2.G), and a TP53 KO plasmid using Lipofectamine 3000 regent according to the manufacturer’s instruction. At 48 h after transfection, the supernatants was collected, filtered through a 0.45 mm filter, and centrifuged at 20,000 rpm for 2 h at 4 °C to harvest virus particles. Virus was immediately added to DLD1 cells with 8 μg/mL polybrene. After infection for 4 days, cells were selected for stable expression in the presence of 3 μg/mL puromycin for one week. The puromycin-resistant stable clones were pooled. The TP53 deficiency was confirmed by Western blotting and quantitative real-time PCR.

### Human tissue samples, organoid culture, and PDX modeling

All human tissue samples who had not been received any chemotherapy or radiotherapy prior to surgery were obtained from Guangxi Medical University Cancer Hospital. All patients provided written informed consent to allow any excess tissue to be used for research studies. The study was approved by the ethics committee of Guangxi Medical University Cancer Hospital.

The organoid culture was performed as described [33]. Briefly, Tissues were minced into small pieces and digested in digestion buffer (200 U/ml type IV collagenase, 125 μg/ml type II dispase, and 50 U/ml deoxyribonucleic I in PBS) at 37 °C for 1 h. Cells were cultured with growth medium (500 ng/mL R-Spondin1, 100 ng/mL Noggin, 40 ng/mL EGF, 20 ng/mL FGF-basic, 10 μM Y-27632, 10 mM Nicotinamide in advanced DMEM/F12) at 37 °C, 5% CO_2_. The organoid growth medium was refreshed every 2 to 3 days.

The establishment of PDX was performed as previously described [34]. Necrotic areas and adipose tissue were removed following surgery. Around 1 mm^3^ of tumor fragments were subcutaneously (s.c.) implanted into the right flank of male NPG mice using a trocar. Successfully engrafted tumor models were then passaged and banked after three passages in mice. After the successful expansion of the F3 generations of two patient-derived colorectal tumor specimens in NPG mice, the F3 PDX fragments were cut in pieces in about 1 mm^3^ and implanted s.c. into the right flank of male NPG mice. Tumor growth was measured every 3 days. Randomly allocate each group of mice before medication. When the tumors reached about 50 mm3, mice were randomly divided into four groups and intraperitoneal (i.p.) injected with the vehicle (PBS), TAS102 (150 mg/kg), SAS (250 mg/kg), or TAS102 (150 mg/kg) combine with SAS (250 mg/kg) every day for 5 days as the first round. Two days later, the mice were managed for a second round of treatment the same as before for another 5 days. Mice weight and tumor volume were recorded every 2 days. Tumor sizes were measured with a caliper, and tumor volumes were calculated using the formula: 0.5 × length × width^2^. Mice were sacrificed 2 days after the second round of treatment, and tumors were collected and photographed.

### Immunohistochemistry

Tissue and cell precipitation were fixed with 4% Paraformaldehyde for 48 h, and then embedded in paraffin to make 4-μ Slice m, perform HE staining and immunohistochemistry staining, take photos under a microscope, and collect immunohistochemical images of each group. Immunohistochemical primary antibodies include Ki67 (Servicebio, GB121141-100), p53 (Servicebio, GB12626-100), and SLC7A11 (Affinity, #DF12509).

### Transcriptomics

After treating RKO cells with TAS102 for 48 h, total RNA was extracted using the Trizol method, and the quality of the extracted RNA was checked using agarose gel electrophoresis. Subsequently, a transcriptome library was constructed by mixing the first-strand reaction buffer with random primers, isolating mRNA, fragmenting and adding primers, synthesizing the first and second strands of cDNA, preparing cDNA library fragment ends, followed by adapter ligation, purification of ligation reaction mixture, and finally PCR library enrichment and purification. The obtained data were subjected to differential enrichment analysis, with Log2FC fold change used as the criterion for differential gene selection, followed by GO functional and KEGG pathway enrichment analysis.

### Proteomics

After treating RKO cells with TAS102 for 48 h, total cellular proteins were extracted, and the protein concentration was determined using the BCA assay kit. SDS-PAGE electrophoresis and Coomassie brilliant blue staining were performed for 1 h, followed by destaining with a destaining solution. The protein bands should be clear, and differences compared to the control group can be observed in the treated samples. Subsequently, the proteins were subjected to reduction, alkylation, acetone precipitation, washing, and enzymatic digestion. The digested samples were taken out, and appropriate desalting columns were used for desalting and peptide quantification according to experimental needs. Finally, spectral databases and DIA data acquisition and analysis (nano-HPLC-MS/MS analysis and data acquisition) were established. Protein annotation was then performed, including GO and KEGG annotation.

### Statistical methods

The data in this study were analyzed using SPSS software (SPSS 23.0, Chicago, Illinois, USA) and GraphPad Prism Version 9.0 software (La Jolla, CA, USA). Continuous numerical variables in both groups were expressed as mean ± standard error of the mean (SEM). Statistical significance for group comparisons was determined using *t*-tests or analysis of variance (ANOVA), and a *p* value <0.05 was considered statistically significant. All in vitro experimental results were obtained from at least three independent biological replicates.

## Results

### FTD/TPI inhibits the vitality and proliferation of CRC cell lines

**Fig. 1:**
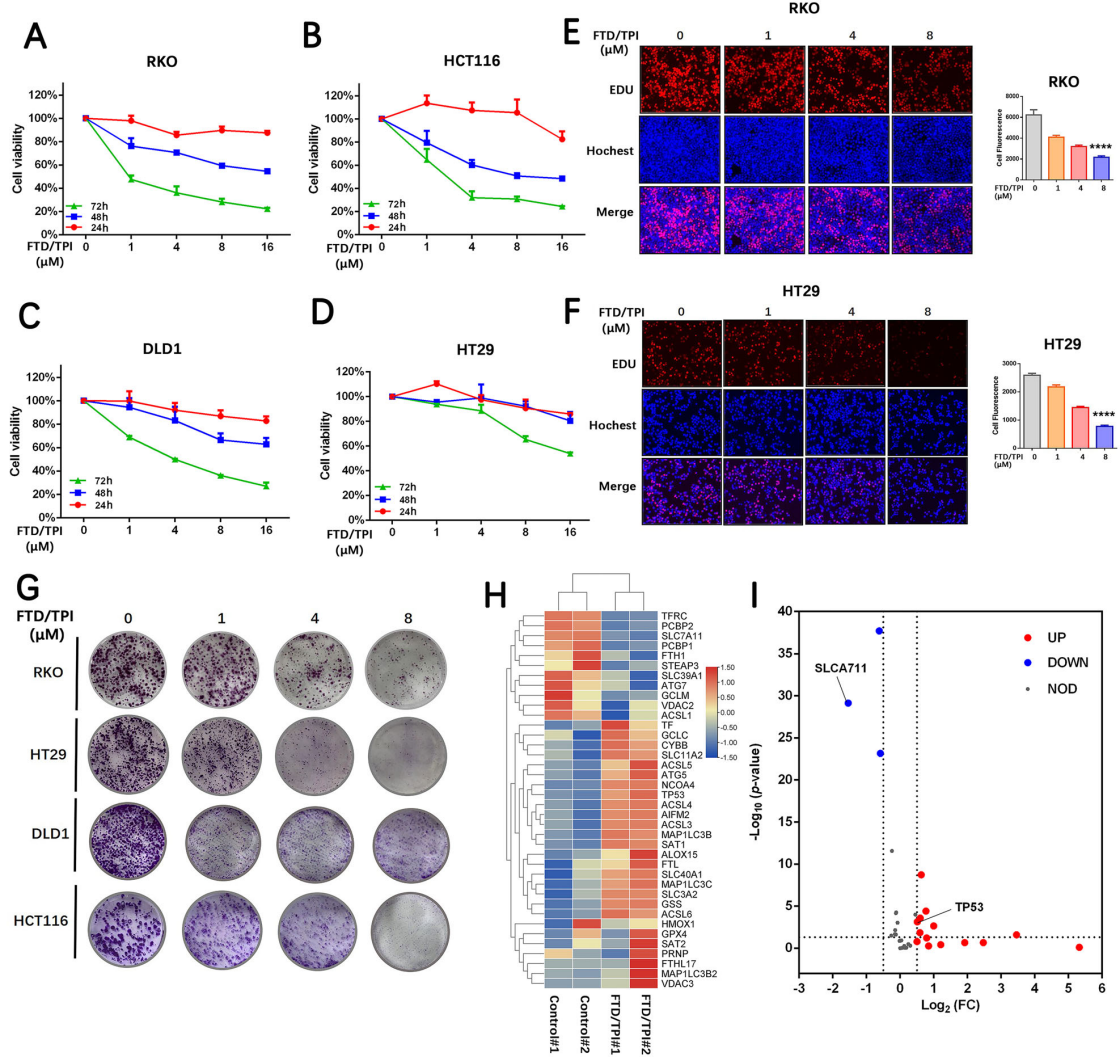
Inhibition of viability and proliferation of colorectal cancer cell lines by FTD/TPI. **A**–**D** Relative cell viability of RKO, HCT116, DLD1, and HT29 cell lines treated with FTD/TPI for 24, 48, and 72 h, with FTD/TPI doses ranging from 0 to 16 μM in a dose-dependent manner. **E**, **F** Fluorescence microscopy images of EDU staining to assess proliferation of RKO and HT29 cell lines after treatment with FTD/TPI at doses of 0, 1, 4, and 8 μM for 72 h. The top row represents red fluorescence, indicating EDU staining, the second row represents blue fluorescence, indicating Hochest staining, and the third row represents merged fluorescence. Scale bar = 100 μm. **G** Clonogenic assay showing the colony-forming ability of RKO, HCT116, DLD1, and HT29 cell lines under the influence of FTD/TPI doses (0, 1, 4, and 8 μM) after 14 days. **H** Heatmap analysis of differentially expressed genes related to the ferroptosis pathway. Red indicates upregulation, while blue indicates downregulation. **I** Volcano plot illustrating differentially expressed genes related to the ferroptosis pathway. Red indicates upregulation, while blue indicates downregulation. Data were presented as representative images or mean values ± SD from three or more independent replicates. Statistical analysis was performed using a two-tailed unpaired *t*-test. *p < 0.05, **p ≤ 0.01, ***p < 0.001, and ****p ≤ 0.0001, indicating statistical significance.

We found that FTD/TPI reduces the viability of four CRC cell lines in a time-dose gradient (Fig. 1A–D). RKO, DLD1, and HCT116 cells exhibited higher sensitivity to FTD/TPI within 24–48 h, whereas HT29 cells showed relatively lower sensitivity in the early stage and required 72 h of treatment for significant inhibition of cell viability. Clonogenic assay after drug treatment also indicated varying degrees of inhibition of colony growth in CRC cell lines by FTD/TPI (Fig. 1G). We selected different concentrations of FTD/TPI to perform EDU fluorescence staining and microscopic imaging on the sensitive cell line RKO and the insensitive cell line HT29. The results showed that high concentrations of FTD/TPI inhibited the proliferation ability of both CRC cell lines (Fig. 1E, F). We also explored the effect of FTD/TPI on cell migration and found that as the time concentration increased, the migration ability of the four cell lines weakened (Fig. S1A–D). When we performed transcriptomic and proteomic sequencing, the sequencing results revealed significant changes in the corresponding genes and proteins within RKO cells after FTD/TPI treatment (Fig. S1E, F).

Cross-analysis of transcriptomics and proteomics data using KEGG pathways revealed the joint effects of the two omics approaches on relevant pathways in cellular biological processes. We found that FTD/TPI treatment regulated cell death through multiple cell death pathways, suggesting that it is a multitarget drug. Interestingly, we discovered that FTD/TPI-treated cells significantly affected molecules related to the ferroptosis regulatory pathway (Fig. S1G). By screening the differentially expressed genes related to ferroptosis in the transcriptomic sequencing results, we identified several transcription factors and target genes affected after drug treatment. Among them, the transcription factor p53 was upregulated, while the SLC7A11 gene was significantly downregulated after treatment with FTD/TPI (Fig. 1H, I).

### FTD/TPI induces CRC cell death

**Fig. 2:**
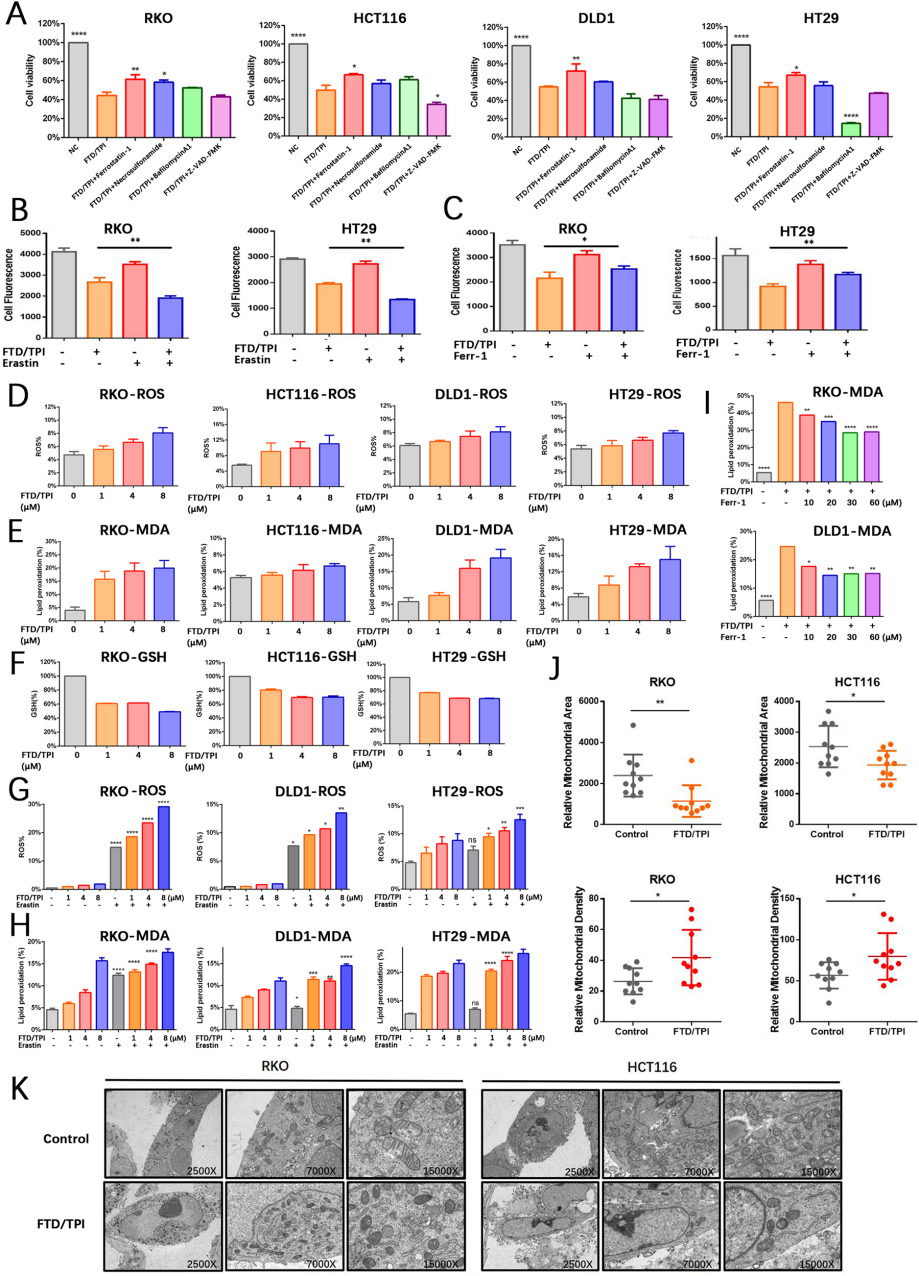
FTD/TPI induces cell death in colorectal cancer cells through ferroptosis. **A** Relative cell viability of RKO, HCT116, DLD1, and HT29 cell lines treated with FTD/TPI (4 μM) in combination with vehicle (DMSO), ferroptosis inhibitor (Ferrostatin-1, 10 μM), necrosis inhibitor (Necrosulfonamide, 1 μM), autophagy inhibitor (BafliomycinA1, 0.5 μM), or apoptosis inhibitor (Z-VAD-FMK, 20 μM) for 48 h. **B** EDU staining to assess proliferation of RKO and HT29 cell lines after treatment with FTD/TPI (0, 1, 4, 8 μM) and 1 μM Erastin for 72 h. **C** EDU staining to assess proliferation of RKO and HT29 cell lines after treatment with FTD/TPI (0, 1, 4, 8 μM) and 2 μM Ferrostatin-1 for 72 h. **D**, **E** Measurement of intracellular reactive oxygen species (ROS) and cellular lipid peroxidation (MDA) levels in RKO, HCT116, DLD1, and HT29 cell lines treated with gradient doses of FTD/TPI (0, 1, 4, 8 μM) for 12 h. **F** Depletion of glutathione (GSH) levels in RKO, HCT116, and HT29 cell lines treated with gradient doses of FTD/TPI (0, 1, 4, 8 μM) for 48 h. **G**, **H** Measurement of intracellular ROS and cellular lipid peroxidation (MDA) levels in RKO, DLD1, and HT29 cell lines treated with gradient doses of FTD/TPI (0, 1, 4, 8 μM) and 1 μM Erastin for 12 h. **I** Measurement of cellular lipid peroxidation (MDA) levels in DLD1 and RKO cell lines treated with 2 μM FTD/TPI in combination with 10, 20, 30, or 60 μM Ferrostatin-1 for 12 h. **J**, **K** Transmission electron microscopy observations of mitochondrial size and mitochondrial membrane density in RKO and HCT116 cells treated with FTD/TPI (8 μM) for 48 h, with quantification at magnifications of 2500X, 7000X, and 15000X. Data were presented as representative images or mean values ± SD from three or more independent replicates. Statistical analysis was performed using a two-tailed unpaired *t*-test. *p < 0.05, **p ≤ 0.01, ***p < 0.001, ****p ≤ 0.0001, indicating statistical significance.

To confirm the primary effects of FTD/TPI on cell death, we performed combination treatment with inhibitors of ferroptosis (ferrostatin-1), necrosis (necrosulfonamide), autophagy (bafilomycin A1), and apoptosis (Z-VAD-FMK) to examine whether the proliferation and viability of CRC cells can be reversed under the inhibition of different pathways (Fig. 2A). The results suggested that the ferroptosis inhibitor ferrostatin-1 significantly rescued FTD/TPI-induced cell death in CRC, whereas the necrosis inhibitors rescued FTD/TPI-induced cell death less significantly. This finding is consistent with previous sequencing results showing that FTD/TPI is involved in multiple signaling pathways. When we next investigated the combination of FTD/TPI and the ferroptosis activator erastin to determine whether it further promotes cell death, we found that it significantly inhibited viability in all four CRC cell lines (Fig. S2B). Also, examining cell proliferation by EDU fluorescence staining of FTD/TPI in combination with erastin or ferrostatin-1, we found that both the sensitive cell line RKO and the insensitive cell line HT29 exhibited similar trends. The combination of FTD/TPI and erastin further suppressed cell proliferation compared with FTD/TPI alone, whereas the combination with eerrostatin-1 significantly restored cell proliferation compared with FTD/TPI alone (Fig. 2B, C and Fig. S2D–G).

Next, we investigated the effects of FTD/TPI in ferroptosis-related functional experiments. The fluorescent probe FerroOrange was used to detect iron ion deposition in colorectal cancer cell lines. It was observed that FTD/TPI treatment dose-dependently elevated the intracellular iron ion deposition in four colorectal cancer cell lines (Fig. S2A). Other related results showed that FTD/TPI treatment led to an accumulation of ROS and lipid peroxidation as well as a decrease in glutathione (GSH) in all four cell lines (Fig. 2D–F), indicating the involvement of the ferroptosis process. FTD/TPI, in combination with erastin, greatly enhanced the extent of ferroptosis in CRC cells, leading to a significant increase in ROS and lipid peroxidation levels (Fig. 2G, H). When FTD/TPI and ferrostatin-1 were administered simultaneously to RKO and DLD1 cells, the accumulation of lipid peroxidation decreased compared with the administration of FTD/TPI alone (Fig. 2I), indicating that FTD/TPI can induce cell death in CRC cells through the ferroptosis pathway.

For further investigation, we treated RKO and HCT116 cells with FTD/TPI for 48 h and observed the changes in mitochondria by transmission electron microscopy. The results showed a variable degree of mitochondrial shrinkage, a significant reduction in mitochondrial volume with a simultaneous increase in membrane density, a high electron density within the membrane, and expanded cristae (Fig. 2K). Quantification of mitochondrial volume and membrane density revealed significant differences after treatment (Fig. 2J). These findings confirm that ferroptosis involved in FTD/TPI treatment triggers cell death in CRC. The combination of FTD/TPI and ferroptosis activators enhances cell death in CRC, which provides the basis for further research into the combination of FTD/TPI and ferroptosis activators as a new therapeutic strategy for the clinical treatment of CRC.

### FTD/TPI induces ferroptosis in CRC through the p53-SLC7A11 axis

**Fig. 3:**
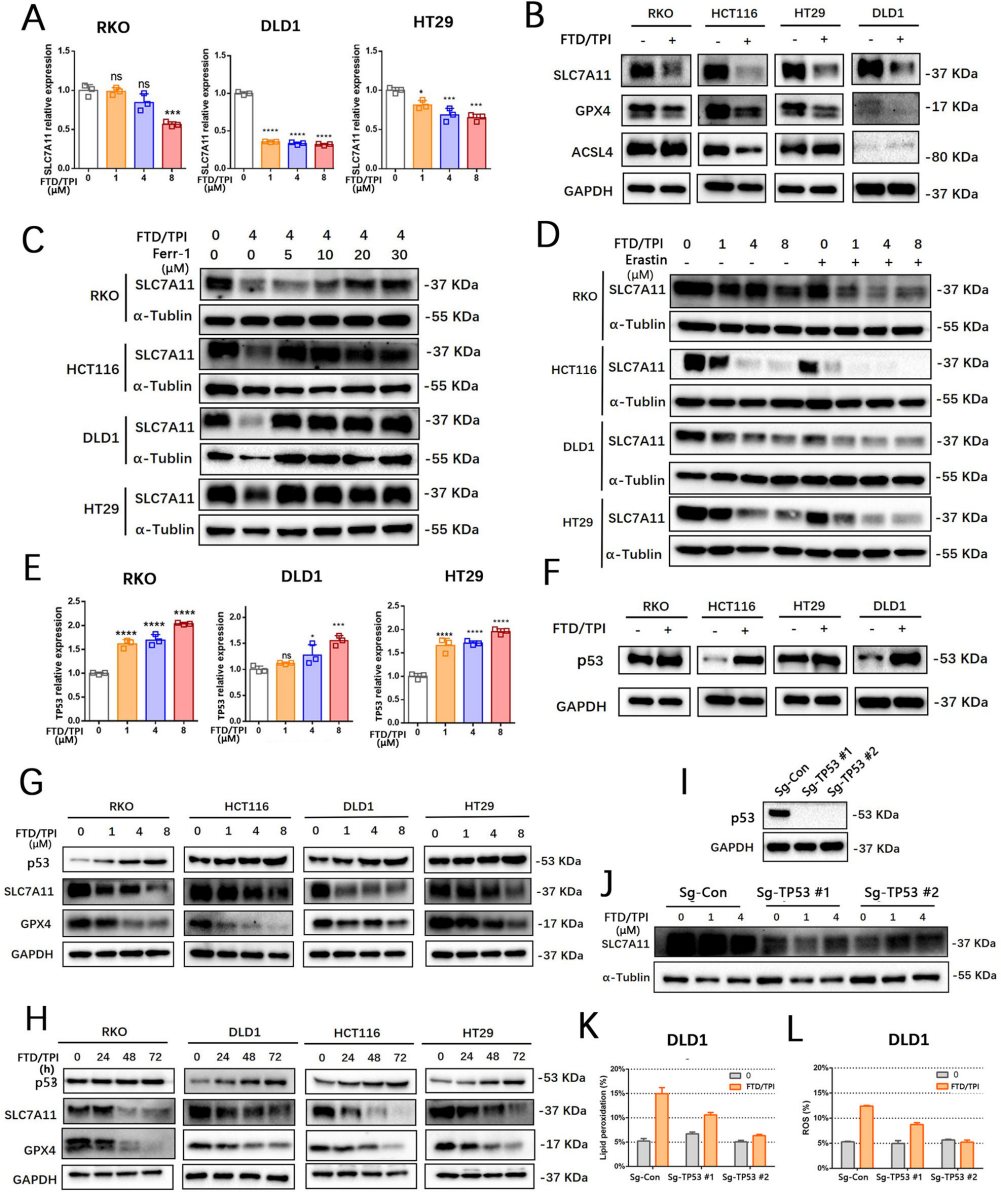
FTD/TPI regulates ferroptosis in colorectal cancer through the p53-SLC7A11 axis. **A** RNA expression of SLC7A11 was assessed at gradient concentrations of FTD/TPI (0, 1, 4, 8 μM) after 48 h of treatment in RKO, HCT116, DLD1, and HT29 cell lines. **B** Expression of ferroptosis-related target protein was detected by Western blotting after treatment with FTD/TPI (4 μM) for 48 h in RKO, HCT116, DLD1, and HT29 cell lines. **C** Protein expression of total SLC7A11 was measured after treatment with 2 μM FTD/TPI in combination with 5, 10, 20, or 30 μM Ferrostatin-1 for 48 h in RKO, HCT116, DLD1, and HT29 cell lines. **D** Protein expression of total SLC7A11 was evaluated after treatment with gradient doses of FTD/TPI (0, 1, 4, 8 μM) and 1 μM Erastin for 48 h in RKO, HCT116, DLD1, and HT29 cell lines. **E** RNA expression of TP53 was assessed at gradient concentrations of FTD/TPI (0, 1, 4, 8 μM) after 48 h of treatment in RKO, HCT116, DLD1, and HT29 cell lines. **F** Expression of p53 was detected by Western blotting after treatment with FTD/TPI (4 μM) for 48 h in RKO, HCT116, DLD1, and HT29 cell lines. **G** Protein expression of p53, SLC7A11, and GPX4 was evaluated at gradient concentrations of FTD/TPI (0, 1, 4, 8 μM) after 48 h of treatment in RKO, HCT116, DLD1, and HT29 cell lines. **H** Protein expression of p53, SLC7A11, and GPX4 was assessed at different time points (0, 24, 48, 72 h) after treatment with FTD/TPI in RKO, HCT116, DLD1, and HT29 cell lines. **I** Western blot detected the p53 protein expression in DLD1 cells with TP53 knockout. **J** Protein expression of SLC7A11 in TP53 knockout DLD1 cells after treatment with FTD/TPI (0, 1, 4 μM) for 48 h. **K**, **L** Comparison of ROS and MDA accumulation levels after treatment with 4 μM FTD/TPI for 12 h in TP53 knockout or wild-type DLD1 cells. Data were presented as representative images or mean values ± SD from three or more independent replicates. Statistical analysis was performed using a two-tailed unpaired *t*-test. *p < 0.05, **p ≤ 0.01, ***p < 0.001, ****p ≤ 0.0001, indicating statistical significance.

The transcriptomic sequencing results indicated that SLC7A11 is involved in FTD/TPI treatment. To verify this observation, we detected the related mRNA and protein expression of SLC7A11. The results revealed that FTD/TPI suppressed the mRNA and protein expression of SLC7A11 in the CRC cell lines (Fig. 3A, B). We also found that use of the ferroptosis antagonist ferrostatin-1 in the FTD/TPI treatment effectively reversed the FTD/TPI-induced suppression of SLC7A11 protein expression, especially in the RKO and DLD1 cell lines (Fig. 3C). Moreover, we observed that the combined use of FTD/TPI and ferroptosis agonist erastin further reduced SLC7A11 protein levels in all four CRC cell lines in a gradient manner (Fig. 3D).

Subsequently, we screened for transcription factors associated with ferroptosis in the four CRC cell lines after 48 h of FTD/TPI treatment. As observed in the previous exploration of bioinformatics, we observed a significant increase in endogenous p53 mRNA and protein expression in all four CRC cell lines (Fig. 3E, F). It is well known that SLC7A11 is a target gene of p53 in the activation of ferroptosis [14, 15]. We hypothesized that SLC7A11 may be the downstream target of FTD/TPI in the activation of p53-mediated ferroptosis. When we examined the expression of the xCT/GPX4 system after treatment, we found a significant decrease in the protein expression of SLC7A11 and GPX4 (Fig. 3G). Evaluating the effects of FTD/TPI at different concentrations and treatment durations on the expression of p53 and SLC7A11, we observed that FTD/TPI effectively promoted p53 protein expression and inhibited SLC7A11 expression in the four CRC cell lines in a dose- and time-dependent manner (Fig. 3G, H). Moreover, we observed that the impact on p53 and SLC7A11 protein expressions in FTD/TPI-treated HT29 cells was relatively minor, which may indicate the response for the viability of FTD/TPI treatment on HT29 cells.

To investigate whether FTD/TPI relies on the transcription factor p53 to regulate the downstream target SLC7A11 expression, we used CRISPR/Cas9 technology to knock out the TP53 gene in DLD1 cells and explore the relationship between the transcription factor p53 and downstream SLC7A11. We selected two DLD1 pool clone cells with p53 protein knockout (Fig. 3I). After treating the TP53 knockout and wild-type cells with a gradient of FTD/TPI, we observed a gradient decrease in SLC7A11 protein expression in the wild-type cells. Although the TP53 knockout cells did not exhibit the same trend, they showed some recovery in SLC7A11 protein expression levels (Fig. 3J). Furthermore, we found that knockout TP53 attenuated the levels of ROS and lipid peroxidation accumulation in the TP53 knockout cells compared with the wild-type cells (Fig. 3K, L). The results provide strong evidence that FTD/TPI induces ferroptosis in CRC through the p53-SLC7A11 axis.

### FTD/TPI directly binds to p53 and enhances p53 protein stability by promoting MDM2 ubiquitination and degradation

**Fig. 4:**
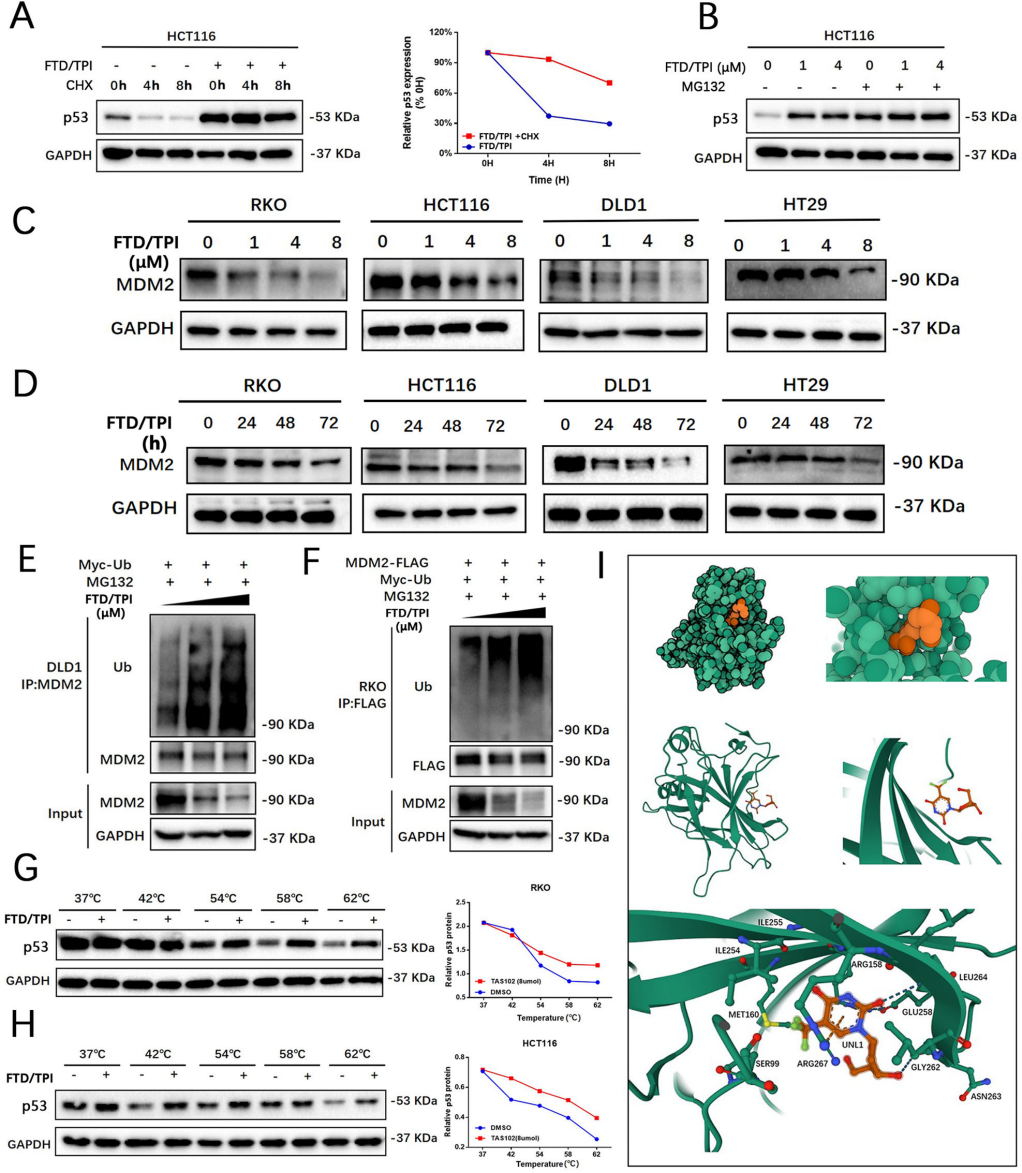
FTD/TPI directly binds to p53 and enhances p53 protein stability by promoting MDM2 ubiquitination and degradation. **A** HCT116 cells were pre-treated with FTD/TPI (4 μM) for 24 h, followed by the addition of 100 μg/mL CHX. Cells were collected at specified time points (0, 4, and 8 h) after CHX treatment, and p53 protein levels were examined by Western blotting. **B** HCT116 and RKO cells were pre-treated with FTD/TPI (0, 1, 4 μM) for 40 h, followed by the addition of 10 μM MG132 for an additional 8 h. p53 protein levels were then assessed by Western blotting. **C**, **D** Protein expression levels of MDM2 were evaluated at different time points (0, 24, 48, 72 h) and doses (0, 1, 4, 8 μM) after FTD/TPI treatment in RKO, HT29, DLD1, and HCT116 cell lines. **E** DLD1 cells transfected with myc-Ub were co-incubated with FTD/TPI (10 μM) at specified concentrations for 40 h, followed by treatment with 10 μM MG132 for 8 h. Cell lysates were immunoprecipitated with anti-MDM2 antibody, and the level of cellular ubiquitination was assessed by Western blotting. **F** RKO cells co-transfected with Flag-tagged MDM2 and myc-Ub expression vectors were co-incubated with FTD/TPI (10 μM) at specified concentrations for 40 h, followed by treatment with 10 μM MG132 for 8 h. Cell lysates were immunoprecipitated with anti-Flag M2 Sepharose, and the level of ubiquitination was examined by Western blotting using specific antibodies. **G**, **H** Melting curves of p53 protein in RKO and HCT116 cells after 1-h FTD/TPI treatment, as determined by CETSA, displaying the quantitative changes in p53 protein at different temperature points based on Western blot analysis. **I** Molecular docking of FTD/TPI drug molecule with p53 (PDB: 8DC8) protein structure, showing the docking site and predicted binding sites of amino acids in the p53 protein. The green represents the p53 protein molecule, and the orange represents FTD/TPI.

To determine how FTD/TPI influences p53 protein in the activation of ferroptosis, we next investigated the regulatory factors of p53. We examined whether FTD/TPI affects the half-life of the p53 protein using cycloheximide (CHX) to inhibit protein translation. The results showed that CHX treatment decreased the expression of p53 protein in HCT116 cells in a time-dependent manner and that the addition of FTD/TPI prolonged the half-life of p53 proteins (Fig. 4A). Treatment with the proteasome inhibitor MG132 enhanced FTD/TPI-induced p53 protein accumulation (Fig. 4B), suggesting that the increased p53 levels may be due to reduced degradation.

MDM2 is an E3 ubiquitin ligase that negatively regulates p53. Therefore, we examined the expression levels of MDM2 protein in the four CRC cell lines after treatment with FTD/TPI. The results showed that FTD/TPI treatment decreased MDM2 expression in all four CRC cell lines in a time- and dose-dependent manner (Fig. 4C, D). Next, we performed ubiquitination experiments using Myc-tagged ubiquitin expression vectors alone or co-transfected with Flag-tagged MDM2 into DLD1 cells. We incubated cells with 5 and 10 μM FTD/TPI for 48 h before treatment with 10 μM of MG132. We subjected the cell lysates to immunoprecipitation using anti-MDM2 antibody or anti-Flag magnetic beads, followed by separation on SDS/PAGE and immunoblotting analysis with anti-ubiquitin antibody. As shown in Fig. 4E, F, FTD/TPI significantly increased the ubiquitination of endogenous and exogenous MDM2.

In a contrasting experiment, we investigated whether FTD/TPI influenced p53 stability by directly binding to p53. Cellular thermal shift assay (CETSA) is primarily used to validate the binding of a molecule to its target protein, a technique widely adapted to drug discovery workflows [35]. In HCT116 and RKO cells, FTD/TPI was able to bind to p53 protein and stabilize it when the temperature increased. The melting curves of p53 protein expression shifted to the right compared with the control group (Fig. 4G, H), indicating a higher expression of p53 protein at the same temperature. This result indicates that FTD/TPI can stably bind to the p53 protein.

To evaluate the potential binding of the p53 protein to FTD/TPI, we assessed FTD (PubChem CID: 6256), the major component of FTD/TPI, and the crystal structure of p53 ((PDB: 8DC8)) for molecular docking analysis. The results revealed that FTD binds to the p53 protein through visible hydrogen bonds and strong electrostatic interactions. We observed that FTD successfully occupies the hydrophobic pocket of the p53 protein structure. The binding energy of FTD to p53 was –6.08 kcal/mol, suggesting a highly stable binding between FTD and p53 (Fig. 4I). The predicted amino acid binding sites for FTD/TPI, and the p53 protein include Ser99, Ile254, Ile255, Met160, Leu264, and Glu258. The docking results indicate that FTD/TPI can effectively interact with two binding sites on the p53 protein. These results suggest that FTD/TPI increases p53 protein stability by directly binding to p53 and promoting ubiquitination and degradation of MDM2, leading to a decrease in the expression of SLC7A11 and GPX4 proteins, which in turn activates ferroptosis.

### SAS enhances FTD/TPI suppression of CRC cells

**Fig. 5:**
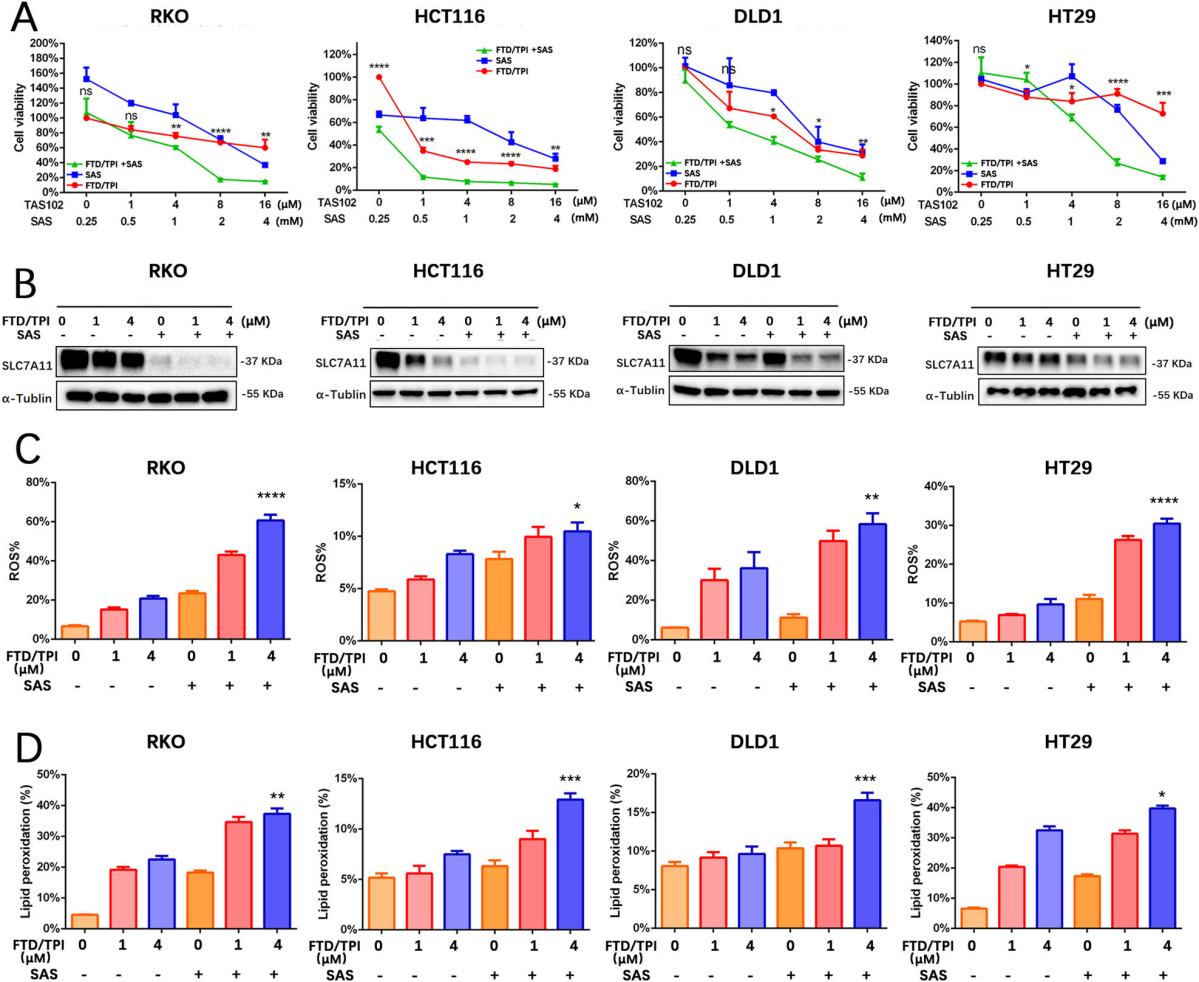
FTD/TPI in combination with ferroptosis inducer sulfasalazine inhibits cell viability in colorectal cancer. **A** Cell relative viability was determined by MTT assay after treatment with FTD/TPI (0, 1, 4, 8, 16 μM) in combination with sulfasalazine (0, 0.25, 0.5, 1, 2, 4 mM) for 48 h in RKO, HT29, DLD1, and HCT116 cells. **B** Protein expression of SLC7A11 was examined by Western blotting after treatment with FTD/TPI (0, 1, 4 μM) in combination with sulfasalazine (1 mM) for 48 h in RKO, DLD1, HCT116, and HT29 cell lines. Data were presented as representative images or mean ± SD from at least three independent replicates. **C**, **D** Accumulation of intracellular reactive oxygen species (ROS) and lipid peroxidation (MDA) levels was measured after treatment with FTD/TPI (0, 1, 4 μM) and sulfasalazine (1 mM) for 12 h in RKO, DLD1, HCT116, and HT29 cell lines. Statistical analysis was performed using a two-tailed paired *t*-test. *p < 0.05, **p ≤ 0.01, ***p < 0.001, ****p ≤ 0.0001, indicating statistical significance.

The results thus far indicate that the combination of FTD/TPI and ferroptosis activators promotes cell death in CRC. To further increase the efficacy of FTD/TPI in clinical treatment, we selected three clinical drugs that have been suggested to be ferroptosis activators for combined treatment with FTD/TPI—SAS, artemisinin (ART) and dihydroartemisinin (DHC) [15, 16]—and performed screening in the sensitive cell line RKO and the insensitive cell line HT29. MTT assays revealed that SAS but not ART or DHC in combination with FTD/TPI treatment resulted in a statistically significant increase in cell death in RKO and HT29 cells (Fig. 5A and Fig. S3A). A noteworthy finding was that the addition of SAS sensitized HT29, HCT116, and DLD1 cells to FTD/TPI (Fig. 5A).

It has been reported that SLC7A11 is the target of SAS in the activation of ferroptosis [14, 15]. To uncover the mechanisms underlying the combination of SAS and FTD/TPI in cell death, we examined the protein expressions of SLC7A11 and GPX4. We found that SAS further promoted the suppression of SLC7A11 and GPX4 in combination with FTD/TPI but that ART or DHC did not (Fig. S3B), which was consistent with the cell viability results. Moreover, the addition of SAS decreased SLC7A11 protein levels induced by FTD/TPI in the four cell lines (Fig. 5B). SAS also promoted the FTD/TPI-induced accumulation of ROS and lipid peroxidation levels within the cells (Fig. 5C, D). These results suggest that SAS synergistically intensifies the killing effect of FTD/TPI in CRC cells, likely by targeting SLC7A11, providing a novel approach for FTD/TPI in the treatment of patients with advanced and CRC without other treatment options.

### FTD/TPI inhibits the vitality and proliferation of CRC organoids

**Fig. 6:**
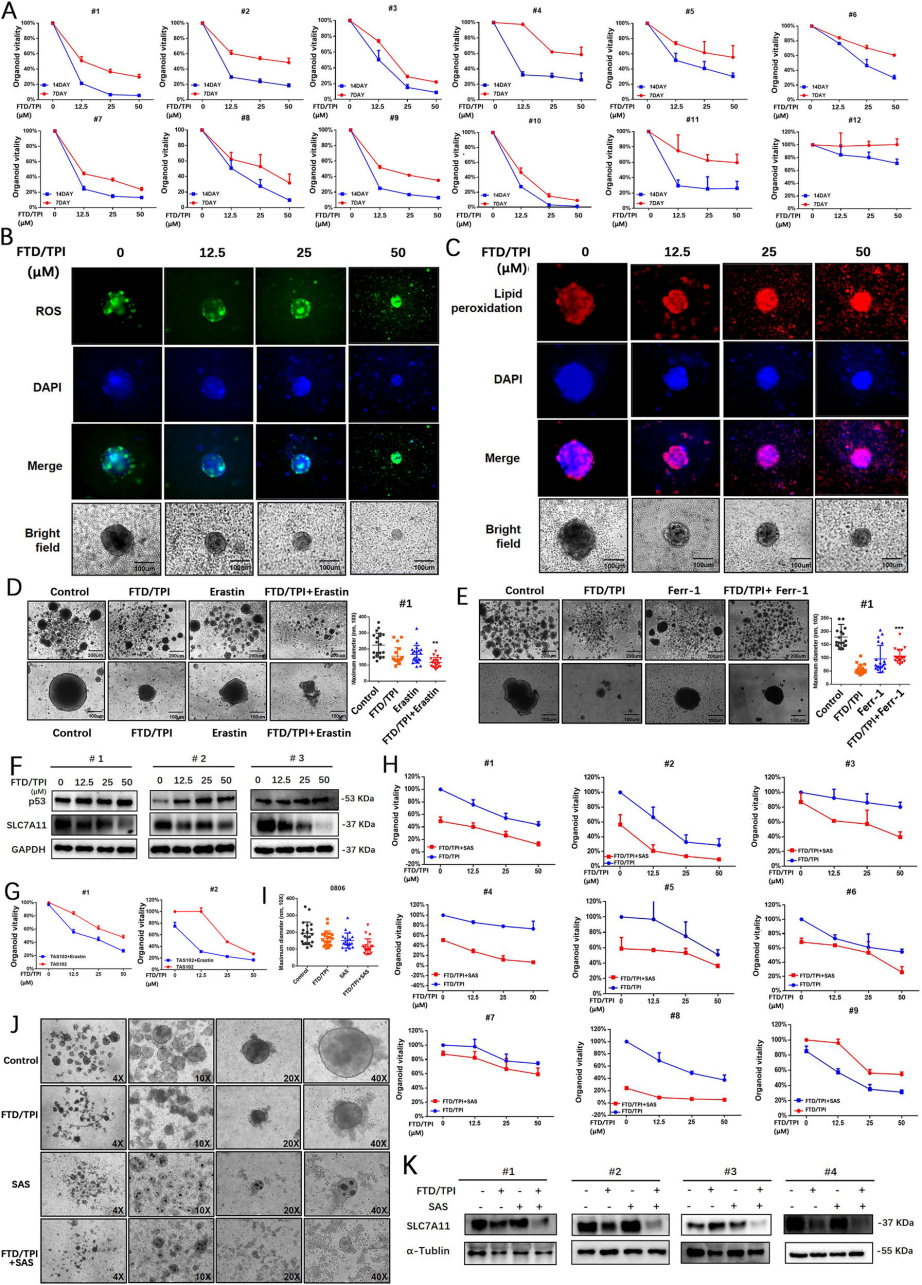
FTD/TPI inhibits organoid viability and proliferation in colorectal cancer. **A** Relative viability of colorectal cancer organoids derived from 12 patients was measured after treatment with FTD/TPI (0, 12.5, 25, 50 μM) for 7 and 14 days. **B**, **C** Accumulation levels of reactive oxygen species (ROS) and lipid peroxidation (MDA) were observed by fluorescence microscopy after treatment with FTD/TPI (0, 12.5, 25, 50 μM) for 7 days in colorectal cancer organoids. The first row represents green fluorescence images for ROS, red fluorescence represents MDA, the second row shows blue fluorescence images for DAPI, the third row presents merged fluorescence images, and the fourth row displays bright-field microscopy images of the organoids. Scale bar = 100 μm, magnification at 20X. **D** Microscopic images of colorectal cancer organoids from three patients were captured after treatment with 12.5 μM FTD/TPI and 1 μM Erastin for 7 days to observe and quantify organoid size. Scale bar = 200 μm, magnification at 4X and 20X. **E** Microscopic image of colorectal cancer organoid from one patient treated with 12.5 μM FTD/TPI and 20 μM Ferrostatin-1 for 7 days to observe and quantify organoid size. Scale bar = 200 μm. **F** Western blot analysis of p53 and SLC7A11 protein expression in colorectal cancer organoids from three patients after treatment with FTD/TPI (0, 12.5, 25, 50 μM) for 7 days. **G** Relative viability of colorectal cancer organoids was measured after treatment with FTD/TPI (0, 12.5, 25, 50 μM) in combination with 2 μM Erastin for 7 days in two patients. **H** Relative viability of colorectal cancer organoids was measured after treatment with FTD/TPI (0, 12.5, 25, 50 μM) in combination with 4 mM sulfasalazine for 7 days in nine patients. **I**, **J** Microscopic images of colorectal cancer organoids from patients were captured after treatment with 12.5 μM FTD/TPI and 4 mM sulfasalazine for 7 days to observe and quantify organoid size. Scale bar = 200 μm, magnification at 4X, 10X, 20X, and 40X. **K** Western blot analysis of SLC7A11 protein expression in colorectal cancer organoids from four patients after treatment with FTD/TPI (12.5 μM) in combination with sulfasalazine (4 mM) for 48 h. Data were presented as representative images or mean ± SD from at least three independent replicates. Statistical analysis was performed using a two-tailed paired *t*-test. *p < 0.05, **p ≤ 0.01, ***p < 0.001, ****p ≤ 0.0001, indicating statistical significance.

To determine whether FTD/TPI suppresses the growth of CRC by inducing ferroptosis, we treated tumor organoids derived from 12 patients with CRC with FTD/TPI (Fig. S4A, B). Assessment of the viability of the 3D organoids showed that the organoids were inhibited by FTD/TPI in varying degrees after 7 or 14 days of treatment in a dose-dependent manner (Fig. 6A). This finding is in line with the clinical response to FTD/TPI in different patients. Hematoxylin and eosin (HE) staining revealed that the morphology of organoids gradually decreased with increasing FTD/TPI dosage, along with a reduction in cell clusters and nuclei (Fig. S4C). Immunohistochemical staining with Ki67 also revealed a decrease in the proportion of Ki67-positive cells in the organoids with increasing FTD/TPI concentration, indicating suppression of cell proliferation (Fig. S4D).

Selecting one of the above organoids (Patient #1) that was sensitive to FTD/TPI to detect ROS and lipid peroxidation, we found that the organoid cell clusters exhibited significant accumulation of ROS and lipid peroxidation fluorescence (Fig. 6B, C). Furthermore, we observed that the morphology of organoid cell clusters affected by FTD/TPI was further destroyed by erastin but rescued by ferrostatin-1 (Fig. 6D, E and Fig. S4E, F). Consistent with this observation, western blot and immunohistochemical staining showed that FTD/TPI increased the levels of p53 protein but decreased SLC7A11 in CRC organoid cells (Fig. 6F). These results suggest that ferroptosis may be involved in FTD/TPI-mediated cell death of CRC organoids via the p53-SLC7A11 axis, which is consistent with previous results in CRC cell lines. Interestingly, the addition of erastin further enhanced the suppression of FTD/TPI in CRC organoid viability (Fig. 6G), suggesting a synergistic effect of ferroptosis activators in FTD/TPI treatment.

Next, we examined the effect of SAS on CRC organoid cells treated with FTD/TPI. As expected, the addition of SAS significantly suppressed the viability of organoids from nine patients treated with FTD/TPI, with the sensitivity varying among the patients (Fig. 6H). Morphologically, a reduction in the size of organoid cell clusters was observed when SAS was combined with the FTD/TPI culture, with the cells assuming a smaller maximum diameter of the spherical shape compared with the other groups and an increased number of scattered cells. A noteworthy finding was that SAS treatment promoted significant aggregation of cell nuclei in the organoid clusters, both alone and in combination with FTD/TPI (Fig. 6I, J), the mechanism of which remains to be further explored. Western blot revealed that the combination of SAS and FTD/TPI further reduced the protein expression of SLC7A11 in CRC organoids, which is consistent with the cell line experiments described above (Fig. 6K). Therefore, the results suggest that FTD/TPI activates ferroptosis in CRC organoids via the p53-SLC7A11 axis, indicating that SLC7A11 may be a target of SAS in enhancing the lethal effect of FTD/TPI on CRC organoids.

### Suppression of FTD/TPI and SAS combination therapy in PDX models

**Fig. 7:**
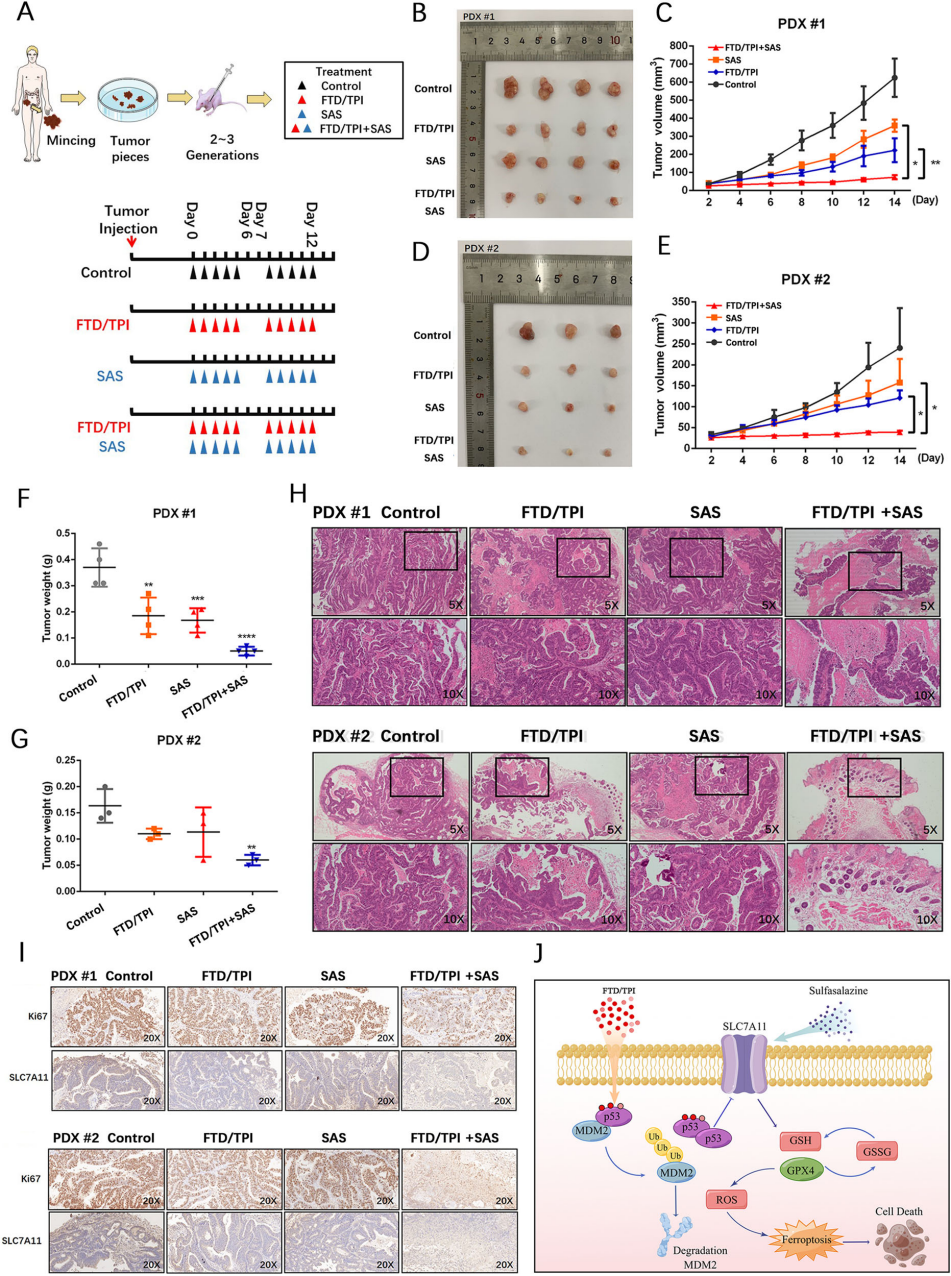
Validating the effectiveness of FTD/TPI in combination with ferroptosis inducer sulfasalazine in PDX models. **A** PDX modeling was performed, followed by organoid culture and passaging for two to three generations before expanding the organoid quantity for grouping. The experimental groups included a control group (physiological saline), a single-agent FTD/TPI group (150 mg/kg), a sulfasalazine group (250 mg/kg), and a combination therapy group. Each treatment group received oral gavage administration. The mice were treated with oral gavage for 1–5 days, left untreated on days 6–7, and continued with oral gavage on days 8–12. Mouse weight and tumor volume were recorded every 2 days, and the experiment lasted for 14 days. **B**–**G** Growth trend graphs of tumor tissues obtained from two PDX models after treatment, as well as quantitative analysis of tumor weight in each group. **H** Histopathological sections stained with hematoxylin and eosin (HE) from each group in two PDX models. **I** Immunohistochemical staining of KI67 and SLC7A11 protein expression in two PDX models. **J** Molecular pathway mechanism diagram. Data were presented as representative images or mean ± SD from at least three independent replicates. Statistical analysis was performed using a two-tailed paired *t*-test. *p < 0.05, **p ≤ 0.01, ***p < 0.001, ****p ≤ 0.0001, indicating statistical significance.

To further validate the efficacy of FTD/TPI in combination with SAS in vivo, we harvested fresh CRC tissues from two patients immediately after surgery. To establish PDX models in nude mice (Fig. S4A), we administered FTD/TPI or SAS to tumor-bearing mice for 5 days per week for 2 weeks (Fig. 7A). We observed that tumors grew much more slowly in the FTD/TPI and SAS combination group than in the control group or the monotherapy groups, with a significant reduction in tumor weight and tumor volume in the combination group (Fig. 7B–G). Moreover, the combination of FTD/TPI and SAS had no effect on body weight or heart, liver, kidney, or lung histology (Figs. S5A–D, S6A, B), indicating that the combination therapy was well tolerated.

Histochemical staining revealed that the tumor tissues of the mice were composed of irregular glandular structures with disordered arrangement, fusion of glands, visible necrosis within the glandular lumens, crowded cell arrangement, enlarged and deeply stained nuclei, increased nuclear-to-cytoplasm ratio, coarse-grained chromatin, and prominent nucleoli (Fig. 7H). Administration of FTD/TPI alone inhibited tumor cell proliferation along with increased expression of p53 protein (Fig. S6C, D). The combination of FTD/TPI and SAS treatment resulted in gland atrophy and shrinkage with a decrease in KI67 and SLC7A11 expression (Fig. 7I). Collectively, these findings suggest that SAS may strengthen the inhibition of FTD/TPI in CRC growth by targeting SLC7A11. Ultimately, we explored that FTD/TPI can promote MDM2 ubiquitination, enhance p53 protein stability, inhibit SLC7A11 expression, and further induce ferroptosis in colorectal cancer cells (Fig. 7J).

## Discussion

In patients with metastatic CRC, only a small percentage can achieve a long-term cure through surgical resection. Outcomes in patients who have previously received multimodal chemotherapy and targeted therapies are suboptimal, with a 5-year survival rate of less than 15% [3]. The novel oral cytotoxic drug FTD/TPI is one of the recommended third-line treatment options for patients with advanced CRC according to the European Society for Medical Oncology (ESMO), the National Comprehensive Care Network (NCCN), and the Japanese Society for Cancer of the Colon and Rectum (JSCCR) guidelines [36–39]. FTD/TPI has been found to confer significant survival benefits and reduce the risk of death and disease progression.

However, the specific targets and mechanisms of cancer biology remain unclear. The results of our study showed that FTD/TPI can activate p53 and suppress transcription and protein expression of downstream SLC7A11, thereby increasing intracellular ROS and lipid peroxidation and inducing iron death in CRC cells. On the one hand, FTD/TPI induces DNA damage, which leads to an increase in TP53 transcription levels. On the other hand, FTD/TPI stabilizes p53 protein by promoting ubiquitination and degradation of MDM2 and by directly binding in various ways. Altogether, our results suggest that the p53-SLC7A11 axis may serve as one of the mechanisms by which FTD/TPI induces iron death for its anticancer effects.

In the four CRC cell lines that we examined, we observed a significant inhibition of cell viability and proliferation after treatment with FTD/TPI, which is consistent with previous reports [38]. To further investigate the targets of FTD/TPI, we performed transcriptomic and proteomic cross-linking analyses after exposing the cell lines to FTD/TPI. We observed a significant increase in the gene expression of the transcription factor p53, which promotes apoptosis and enriches the p53 signaling pathway. This observation is in line with previous reports on apoptosis [8]. By contrast, we also observed that FTD/TPI promoted the enrichment of the ferroptosis signaling pathway accompanied by a decrease in the expression of the cysteine/glutamate antiporter (SLC7A11). To further investigate this observation, we introduced inhibitors of different forms of cell death during FTD/TPI treatment. We found that the ferroptosis inhibitor ferrostatin-1, together with the apoptosis inhibitor, significantly restored the viability of CRC cells treated with FTD/TPI. Conversely, the addition of the ferroptosis activator erastin suppressed cell viability. Electron microscopy revealed noticeable mitochondrial contraction, and at the functional level, FTD/TPI enhanced ferroptosis, as evidenced by a significant increase in ROS and malondialdehyde (MDA) levels in conjunction with a decrease in GSH levels. These results suggest that ferroptosis may be one of the anticancer mechanisms of FTD/TPI in CRC.

Characterized by iron dependence, accumulation of ROS, and lipid peroxidation, ferroptosis represents a distinct form of programmed cell death compared with other cellular death modalities, such as apoptosis. As a heterogeneous tumor, colorectal carcinoma is involved in ferroptosis throughout its development and plays a crucial role in chemotherapy resistance, radiosensitization, and selection of targeted therapies [39–41]. Regulation of ferroptosis in CRC involves multiple targets, with the transcription factor p53 being not only a well-known apoptosis regulator but also a recently confirmed key regulator of ferroptosis. Studies have shown that p53 inhibits cysteine uptake by suppressing SLC7A11 expression, rendering cells susceptible to ferroptosis, and can modulate cysteine metabolism and regulate ROS responses to control ferroptosis [42]. Almost half of CRC patients have p53 missense mutations of varying degrees, leading to single amino acid substitution and contributing to cancer development [43]. Xie et al. have shown that activation of p53 triggers ferroptosis and that TP53 can limit erastin-induced ferroptosis by blocking dipeptidyl peptidase-4 (DPP4) activity in a transcription-independent manner [20]. p53 can also increase sensitivity to ferroptosis by directly inhibiting NRF2 function, rendering tumors with p53 mutations susceptible to oxidative damage and maintaining very low SLC7A11 levels [44]. Therefore, targeting ferroptosis through p53 is a promising strategy for the treatment of CRC that offers a novel approach to inhibiting tumor cells and overcoming chemotherapy resistance in patients with advanced CRC [45].

To further elucidate the effects of FTD/TPI in targeting the p53-SLC7A11 regulatory axis on ferroptosis in CRC cells, we utilized CRISPR/Cas9 to construct TP53 knockout cell lines. Although the addition of FTD/TPI did not lead to further inhibition of the relevant expression levels of the SLC7A11 protein, the addition of MG132 and CHX to FTD/TPI effectively suppressed p53 protein synthesis and prolonged its half-life. Our in vitro immunoprecipitation experiments indicated that the increased ubiquitination of the negative regulatory protein MDM2 may be responsible for the increased expression of p53 protein, whose accumulation was promoted by FTD/TPI. MDM2, which functions as an E3 ubiquitin ligase and negative regulator, promotes multiubiquitination and nuclear degradation of p53 at high levels. By contrast, low levels of MDM2 induce monoubiquitination and nuclear export of p53, maintaining p53 at a lower level but allowing it to fully exert its anticancer function within the cell [46, 47]. Therefore, in cancers associated with p53, releasing p53 from its negative regulators, especially inhibition of MDM2, is critical to releasing the full activity of p53. Xiao-Xin Sun et al. demonstrated that 5-FU stabilizes and activates p53 by disrupting feedback inhibition of MDM2 through blockade of ribosomal protein, specifically that 5-FU treatment stabilizes p53 expression by inhibiting MDM2-mediated ubiquitination and degradation of p53 [48]. The main active ingredient of FTD/TPI, FTD can induce damage and breakage of double-stranded DNA by affecting the cell cycle (G2 phase, S phase) and doping with substances such as DNA strand synthesis, further inhibiting the growth of colorectal cancer cells. This process is not related to the mutation status of p53, and both mutant p53 and wild-type p53 have similar killing effects on colorectal cancer cells [49, 50]. FTD can act on colorectal cancer cells with mutant p53 (DLD1, HT29) or wild-type p53 (HCT116 and RKO), and has varying degrees of cytotoxicity. Moreover, we speculate that regardless of the status of p53, FTD/TPI may participate in MDM2 ubiquitination to enhance the stability of the p53 protein, further inhibiting the expression of SLC7A11 and inducing ferroptosis.

We also performed cellular thermal shift assay (CETSA) experiments and molecular docking to test the prediction that p53 is a direct target of FTD/TPI. We observed that the molecular docking binding energy reached –6.065, and we predicted that FTD/TPI would bind to Ser99, Ile254, Ile255, Met160, Leu264, and Glu258, amino acid binding sites of p53 protein. Amino acid residues such as Ile254 and Met160 in p53 are part of the β-chain in the DNA-binding structural domain involved in the β-folding of the protein, a critical structural element that controls protein function [51]. Ile254-surrounding peptides are considered the driving sequences for the rapid aggregation of oncogenic p53 mutants in the β-chain of the DNA-binding domain. These peptides can lead to cell death much more rapidly than the expected transcription-dependent apoptosis mediated by p53 [52]. When a mutation occurs in the DNA-binding domain of p53 (Glu258), coupling with 4-Hexylresorcinol (4HR) leads to a structural alteration of p53 Ala-258, similar to p53 Glu258. This alternation increases the p53 transcriptional activity of p53 and the expression levels of apoptosis-related proteins [53]. The Ile255 residue may be involved in the core double mutation of the DNA-binding domain of p53 and increases the stability of p53 by increasing the flexibility of the protein [54].

Phosphorylation of the amino acid residue Ser99 near the protein nuclear localization sequence of p53 by protein kinase C (PKC) near the BOX-I domain enhances binding with HDM2 and subsequently induces p53-dependent apoptosis [55]. The N-terminal BOX-I structural domain of p53 contains the major docking site for MDM2. A mutation in the conserved amino acid Leu264 in the S9-S10β-fold region of this structure can stimulate the activity of p53 and increase p53-dependent MDM2 ubiquitination [56]. Based on the current progress of research and the predicted target protein p53, certain amino acid residues involved in binding with FTD/TPI may be involved to some extent in stabilizing p53, such as Leu264, Ile255, and Ser99, and thereby affect ubiquitination of MDM2, which confirmed our conclusion. Our study was the first to observe that FTD/TPI targets p53 as one of its mechanisms, but the manner in which FTD/TPI affects the stability of p53 by binding to its amino acids needs further investigation.

SLC7A11, a downstream target of FTD/TPI, shows a significant reduction in protein expression when treated with FTD/TPI. SLC7A11 is closely associated with microsatellite instability, metastatic potential, and poor prognosis of CRC [57, 58]. Colorectal stem cells are more sensitive to ferroptosis, and knockout of SLC7A11 reduces the levels of cysteine and GSH, thereby inhibiting the viability of CRC stem cells [59]. Furthermore, iron exposure induces ROS to promote the Warburg effect and ferroptosis in CRC cells while suppressing SLC7A11 protein expression [60]. SAS, a multitarget anti-inflammatory drug, can target the Xc^–^transporter protein, disrupt the synthesis of GSH and significant accumulation of ROS, induce ferroptosis, and inhibit growth in CRC cell lines [61]. SAS can also trigger the production of hydroxyl radicals (OH), which are involved in the Fenton reaction, and inhibit GPX4, which together induce ferroptosis. By contrast, normal colonic epithelial cells are less sensitive to SAS [62]. Using FTD/TPI in combination with SAS further reduces viability in CRC cell lines. Microscopically, the combination of FTD/TPI and SAS causes a significant morphologic reduction of CRC organoids, disrupting spherical morphology and increasing the scattering of cells.

We successfully validated the efficacy of the combination therapy by showing that it could reduce ferroptosis to some extent in a PDX murine model. We observed good tolerance to the combined use of the two drugs and no significant toxic changes in the heart, liver, lung, or kidney tissues nor any significant changes in body weight, indicating the feasibility and clinical significance of the therapies. In addition, we observed that SAS-treated cells exhibited deep staining and aggregation of nuclei within cell clusters, a phenomenon not observed with FTD/TPI. This suggests that SAS may have other pathways in its anticancer mechanism beyond ferroptosis induction.

SAS has shown to be a valuable drug targeting the cystine/glutamate antiporter, demonstrating reduced cancer cell proliferation, colony formation, migration, and invasion in vitro [63]. Its therapeutic effects in cancer also include activation of apoptosis-related caspase-3 and the ERK signaling pathway [64]. In CRC, SAS exhibits a unique interaction with KRAS/MMP7/CD44 oncogenes, targeting specific mutation sites and inhibiting the stemness and metastasis of CRC [65]. SAS has been identified as an effective sensitizer for chemo- and radiotherapy in CRC [66]. Therefore, the combination of SAS and FTD/TPI to promote cell death in CRC represents a multi-pathway and multitarget approach to cancer control whose specific mechanism of combination therapy now requires further investigation.

Initial investigations have been conducted into novel ferroptosis-based therapies for CRC. Aspirin has been observed to attenuate lipid formation mediated by the monounsaturated fatty acid SCD1 and thereby act synergistically with RSL3 to jointly promote ferroptosis in CRC cells in a xenograft mouse model [28]. Metformin has been reported to inhibit FAM98A in colorectal cells and downregulate xCT translation, thereby abolishing resistance to the sensitizing agent 5-FU [67]. The combined administration of dihydroartemisinin and iron pyrophosphate has exhibited in vitro cytotoxicity against colorectal tumor cells through an iron-dependent mechanism of ferroptosis [68]. In patients with KRAS-mutant CRC, cetuximab has been shown to inhibit the Nrf2/HO-1 signaling pathway, promoting ferroptosis induced by RSL3 [69]. These findings represent a promising option for patients with advanced CRC, for whom there are few therapeutic alternatives.

The combination of the cytotoxic drug FTD/TPI with ferroptosis activators may be a synergistic therapeutic candidate with multiple targets. We propose viewing the therapeutic application of ferroptosis from another perspective: avoiding the use of ferroptosis inhibitors and antioxidants during FTD/TPI treatment to maximize its efficacy, making ferroptosis a novel approach in CRC therapy. Therefore, gaining a further understanding of the complex molecular regulatory mechanisms underlying CRC resistance and the association of ferroptosis with this process requires experimental investigation. The development of ferroptosis-related drug delivery systems should be explored to share the results of anticancer findings with patients and establish targets for ferroptosis-related biomarkers to predict treatment response and resistance in patients with CRC. Although challenging, targeting ferroptosis and ferroptosis-related biomarkers appears to be a promising approach for the treatment of CRC.

For the first time, we indicated a potential link between FTD/TPI-induced ferroptosis via the p53-SLC7A11 axis and an increase in the stability of the p53 protein through FTD/TPI binding. In addition, we validated the synergistic effect of SAS with FTD/TPI in further inhibiting CRC growth. By exploring the anticancer mechanism of FTD/TPI ferroptosis therapy, we expanded the spectrum of anticancer pharmacology.

## Supplementary information

Western Blot of Manuscript

Supplementary Materials (clean version)

## Data Availability

The original contributions presented in the study are included in the article's supplementary material. Further inquiries can be directed to the corresponding author. All authors agreed on the manuscript.
