# Supplementary material for: Trifluridine/tipiracil induces ferroptosis by targeting p53 via the p53-SLC7A11 axis in colorectal cancer 3D organoids
Source: Cell Death Dis. 2025 Apr 5;16(1):255. doi: 10.1038/s41419-025-07541-z (PMC11972347; doi:10.1038/s41419-025-07541-z)

**Supplementary Materials for**

**Trifluridine/Tipiracil induces ferroptosis by targeting p53 via the p53-SLC7A11 axis in colorectal cancer 3D organoids**

Maosen Huang^1#^, Yancen Wu^1#^, Linyao Cheng^1^, Lihua Fu^1^, Xiaoxia Wei^1^, Haochao Yan^1^, Wene Wei^1,2,4^, Bo Li^5^, Haiming Ru^1,2,3^, Xianwei Mo^1,2,3^, Weizhong Tang^1,2,3^, Zijie Su^1,2,4#^ and Linhai Yan^1,2,3#^

**Corresponding author:**

Linhai Yan, Department of Gastrointestinal Surgery, Guangxi Medical University Cancer Hospital. Nanning, 530021, Guangxi Zhuang Autonomous Region China.

Email: [yanlinhai000@163.com](mailto:yanlinhai000@163.com), Phone: 0086-771-10421

Zijie Su, PhD, Department of Experimental Research, Guangxi Medical University Cancer Hospital. Nanning, 530021, Guangxi Zhuang Autonomous Region China.

Email: [zijiesu@126.com](mailto:zijiesu@126.com), Phone: 0086-771-10421

^#^ These authors contributed equally to this work.

**Original uncropped WB images**
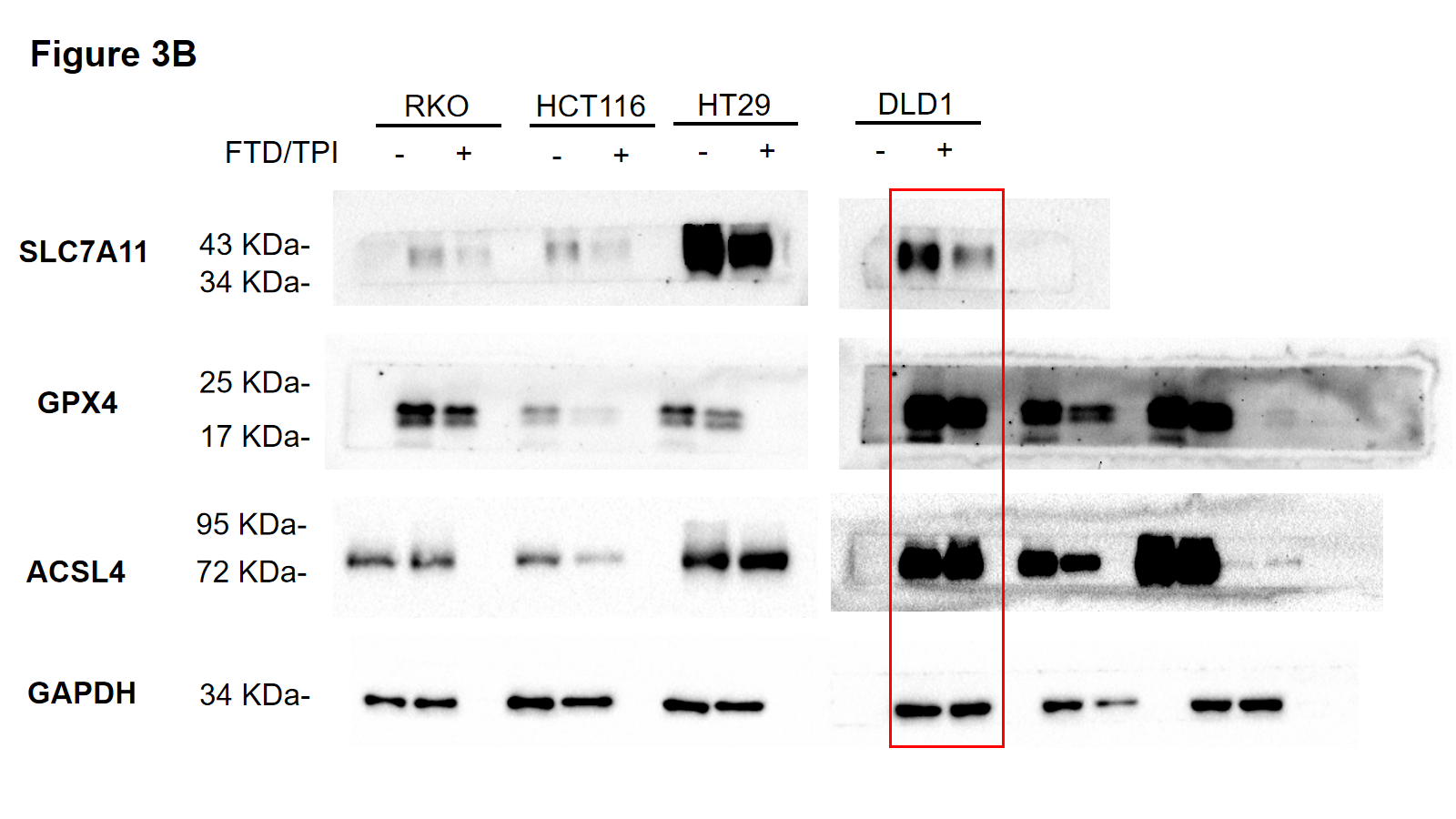

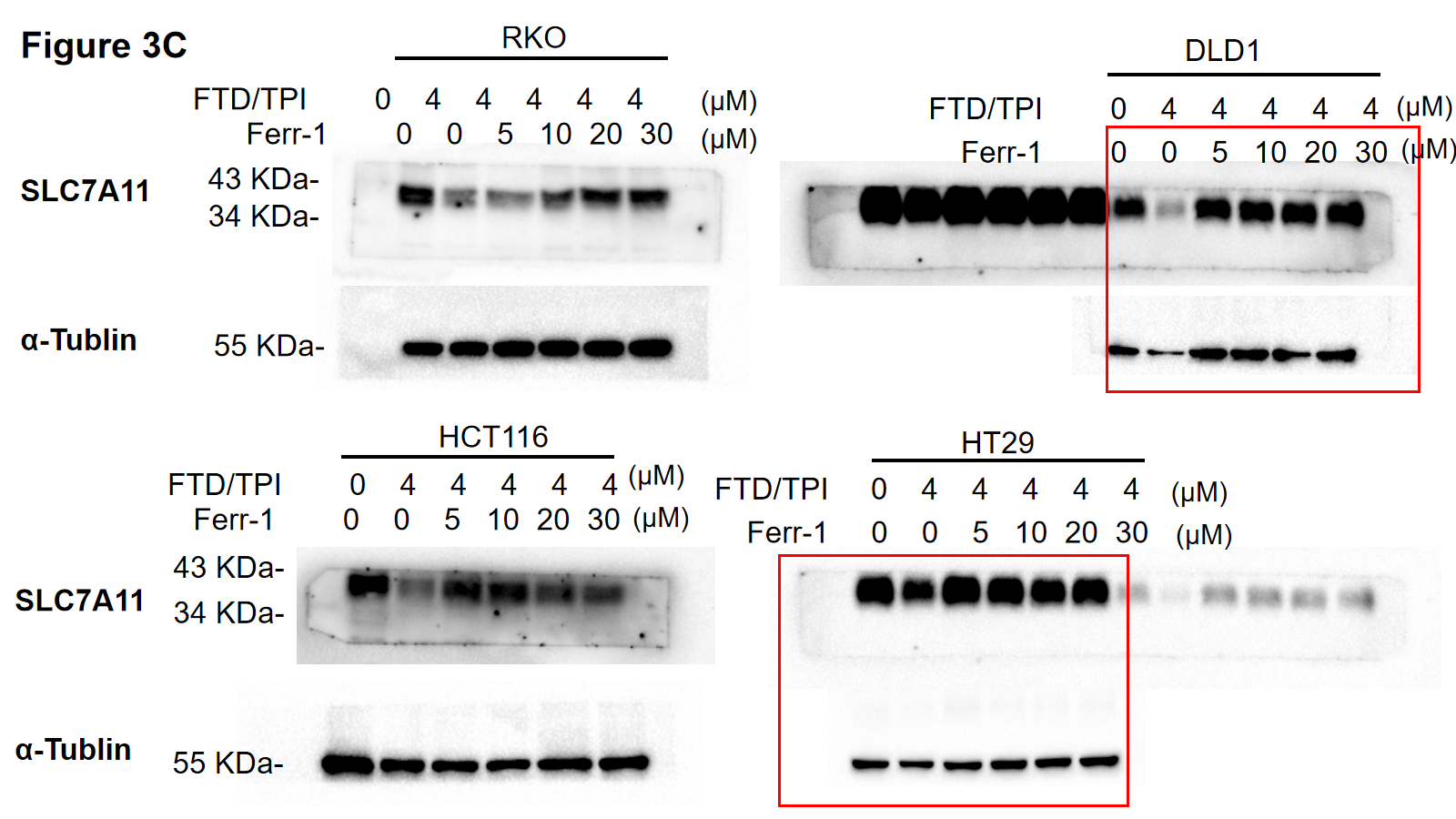

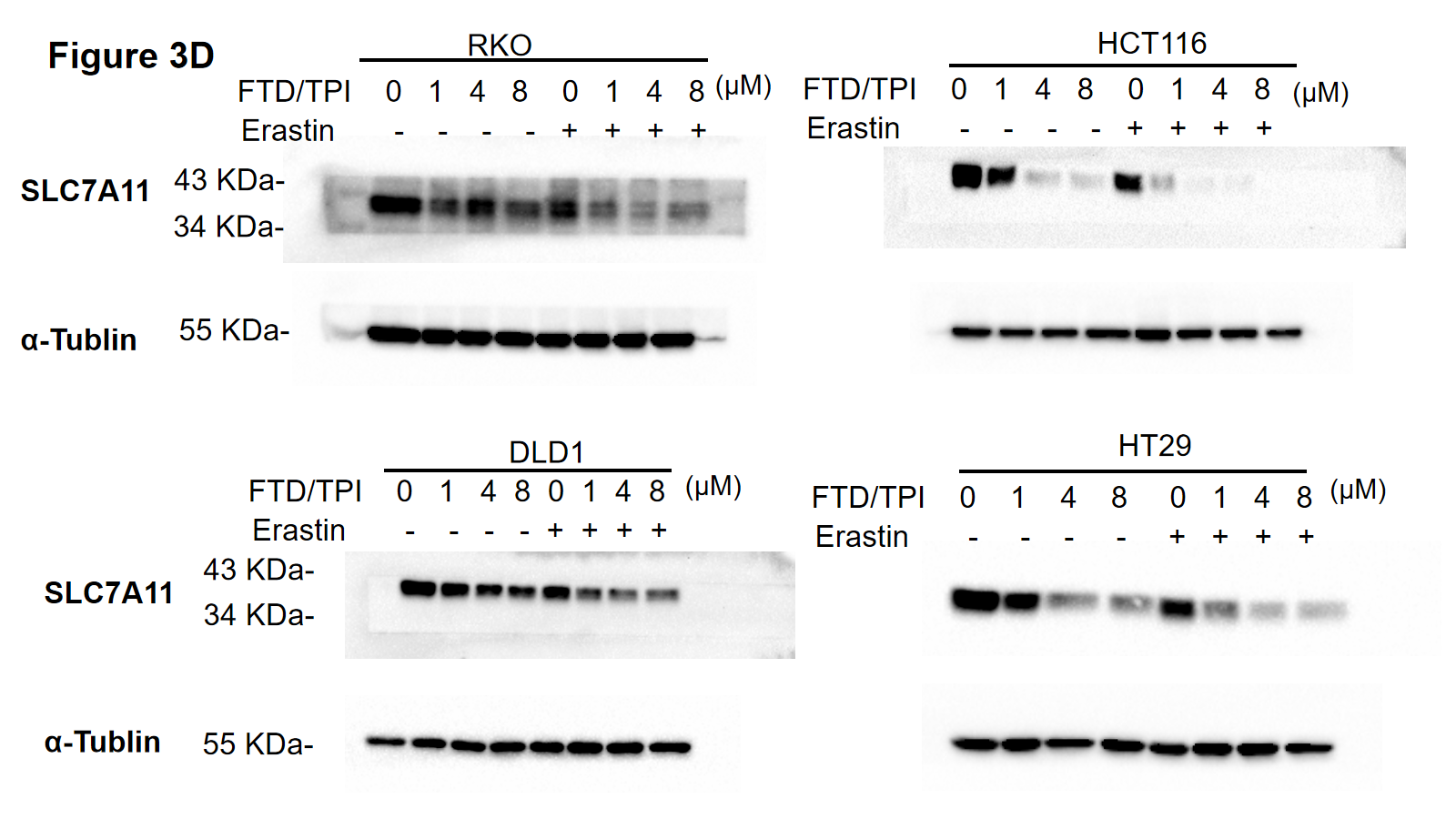

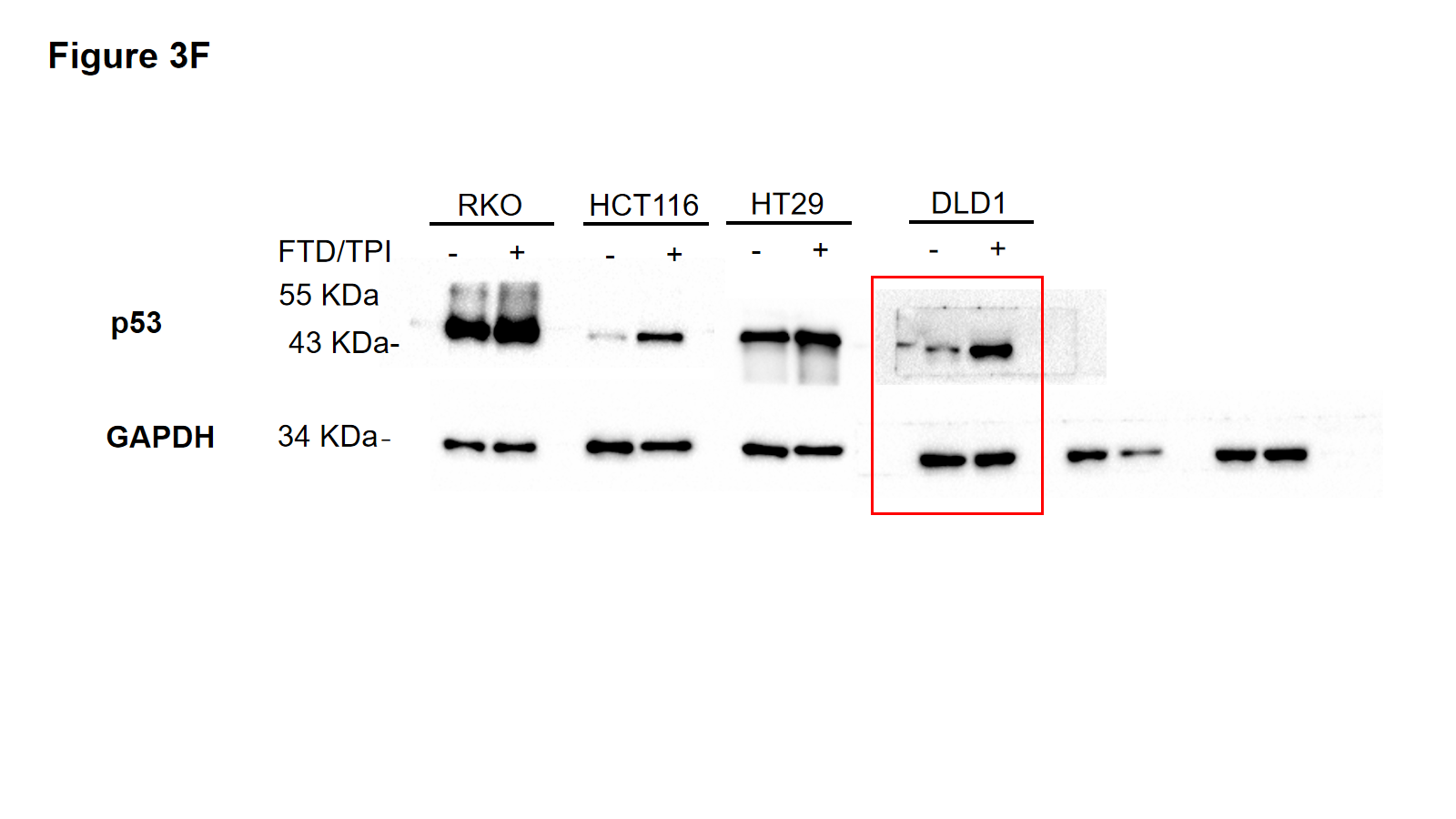

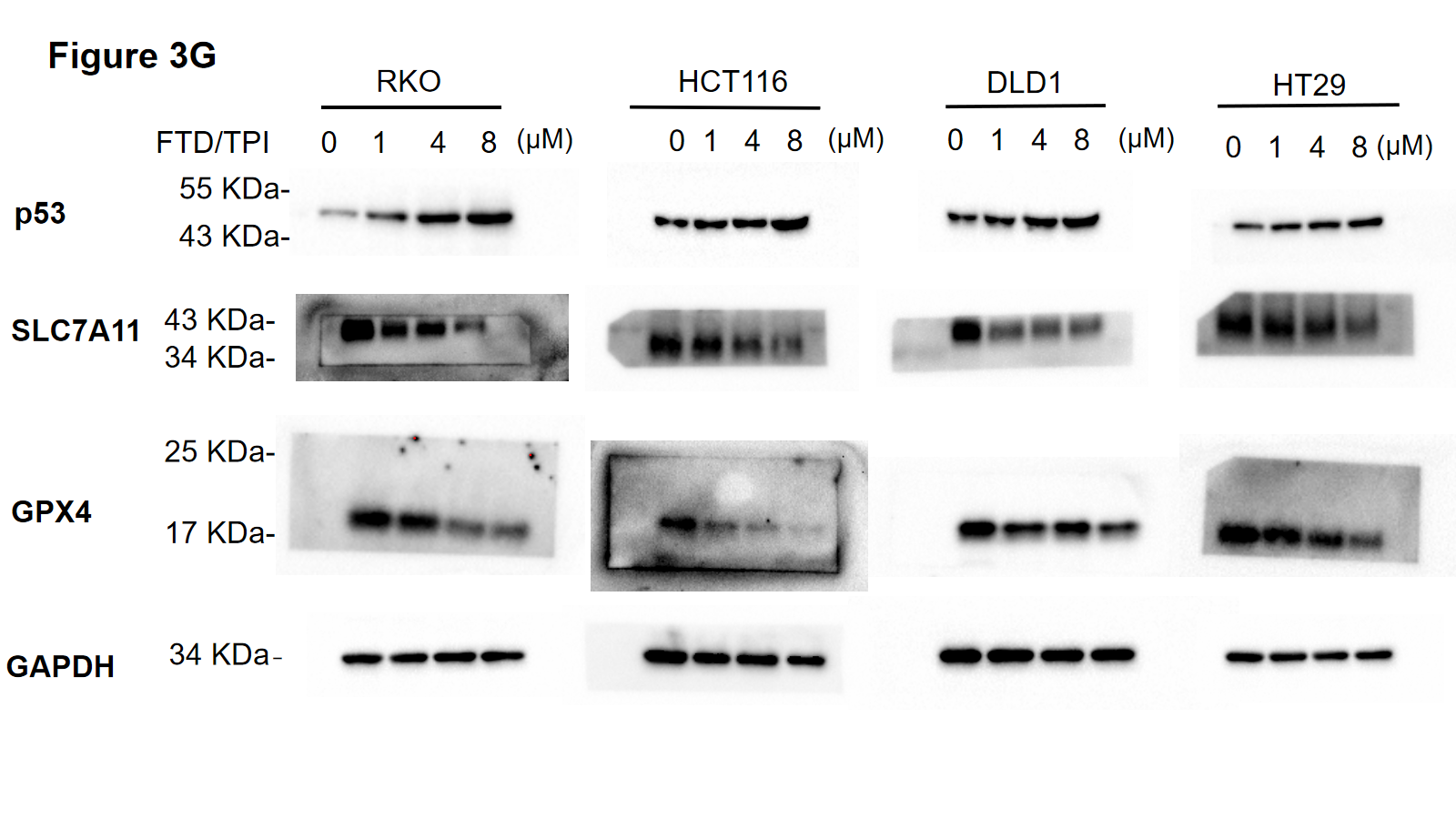

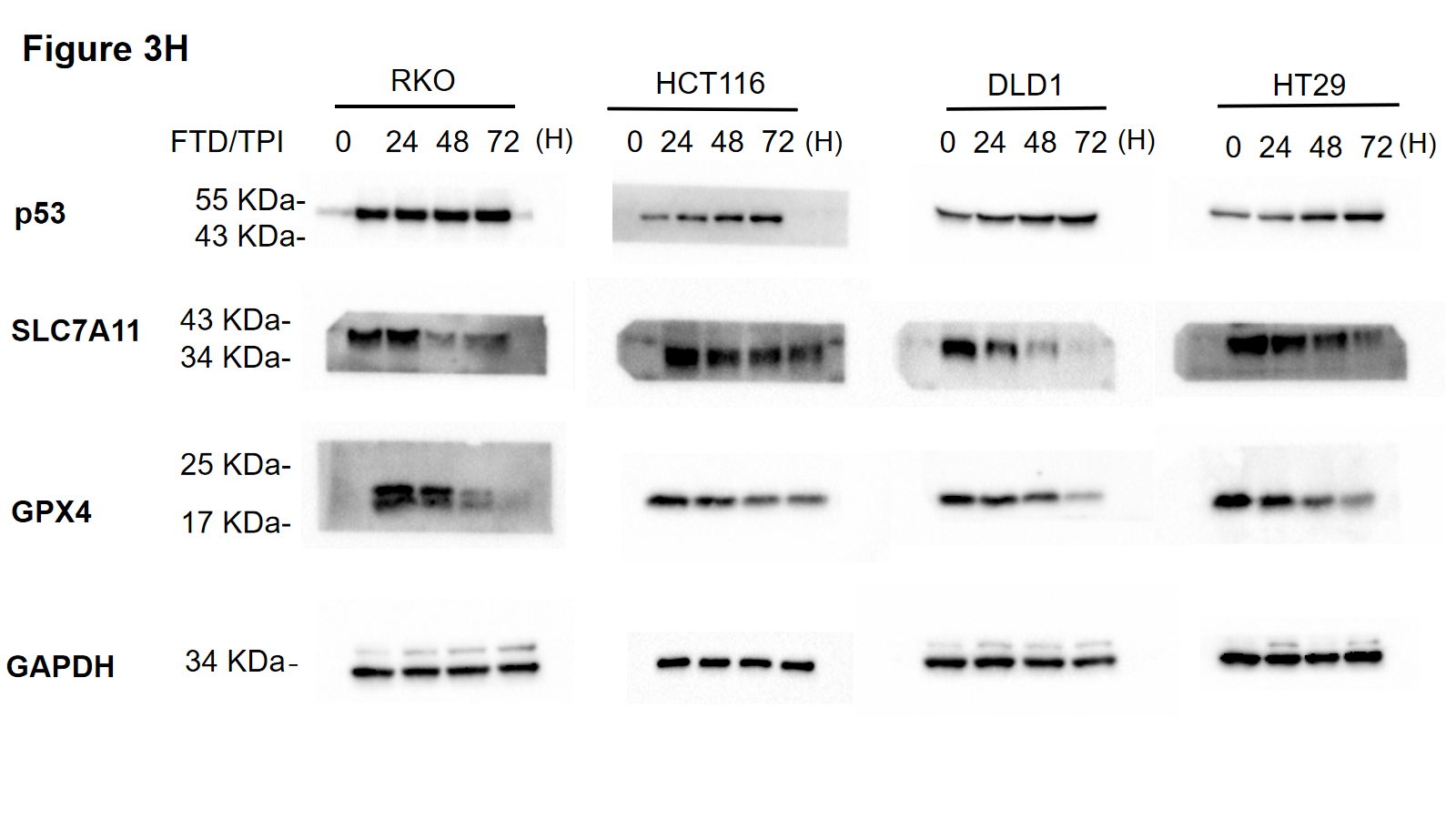

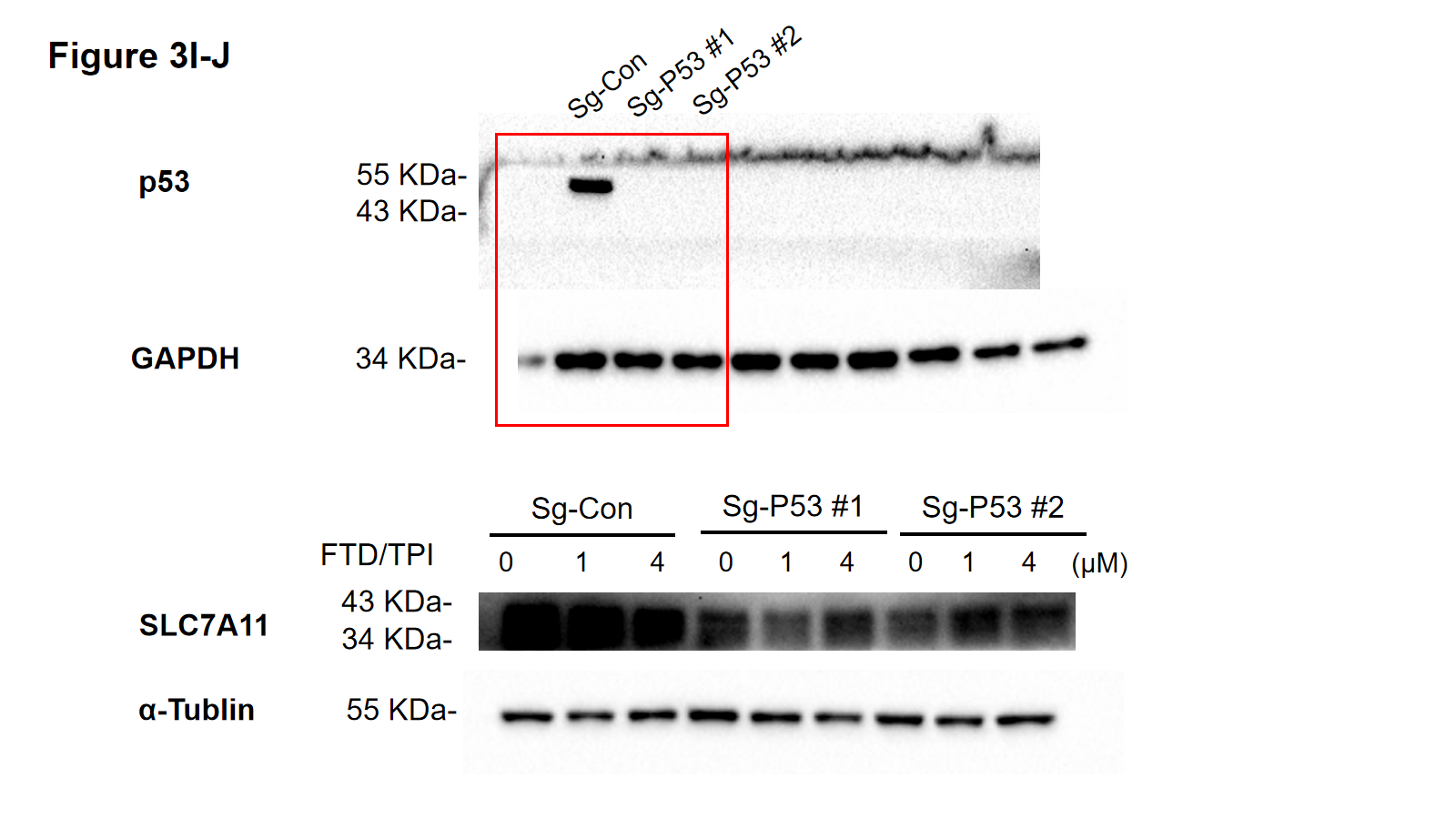

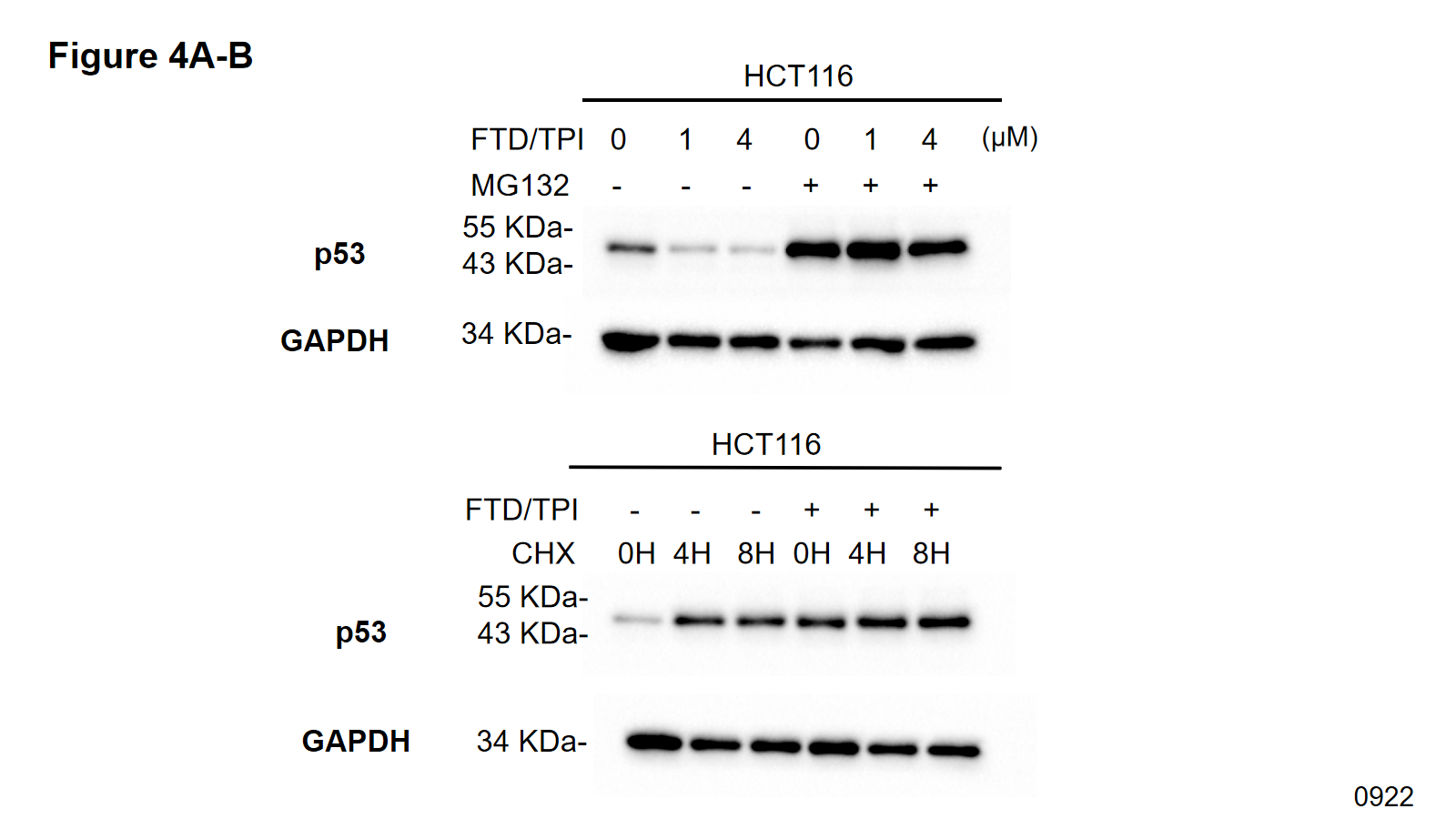

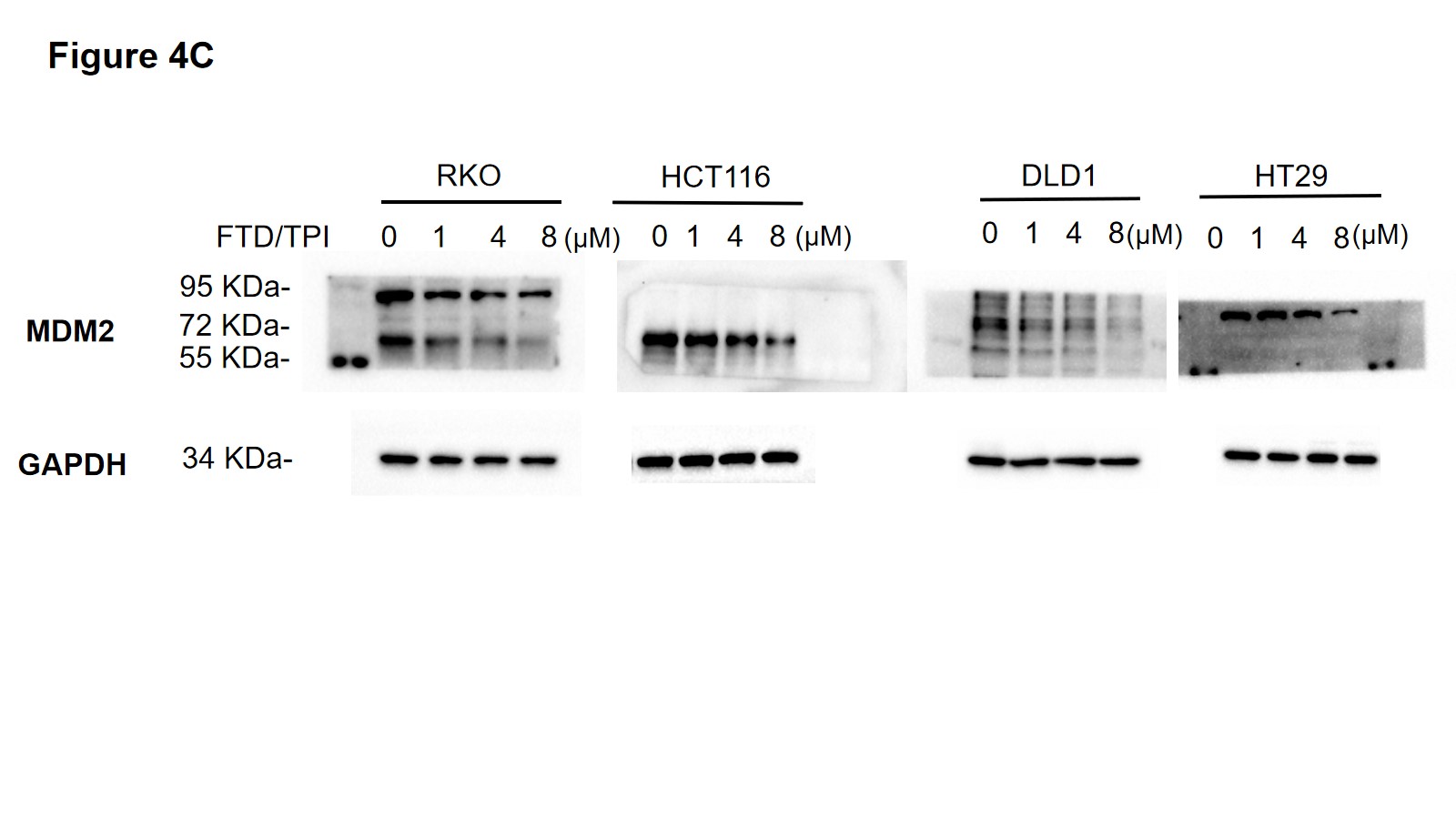

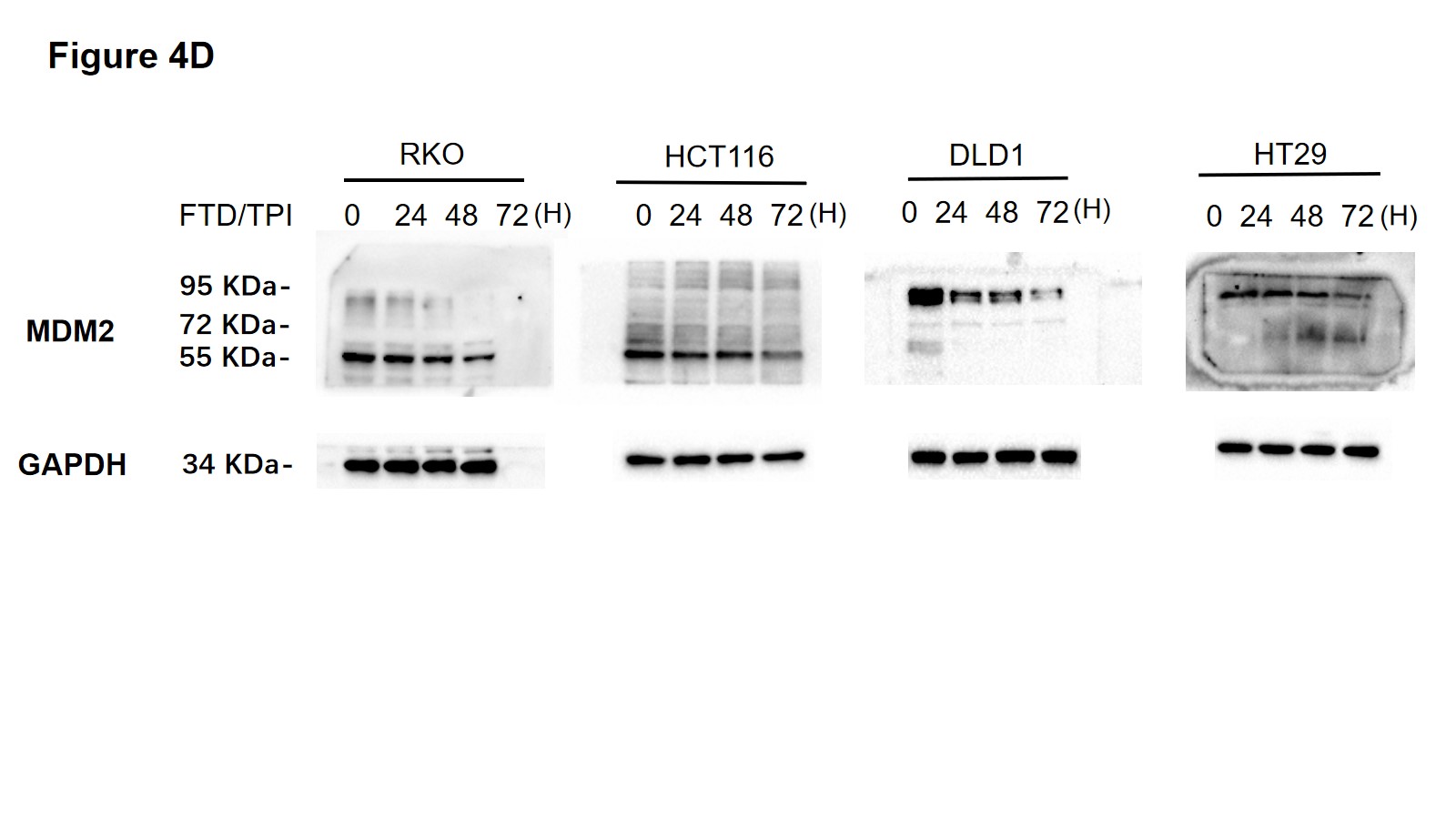

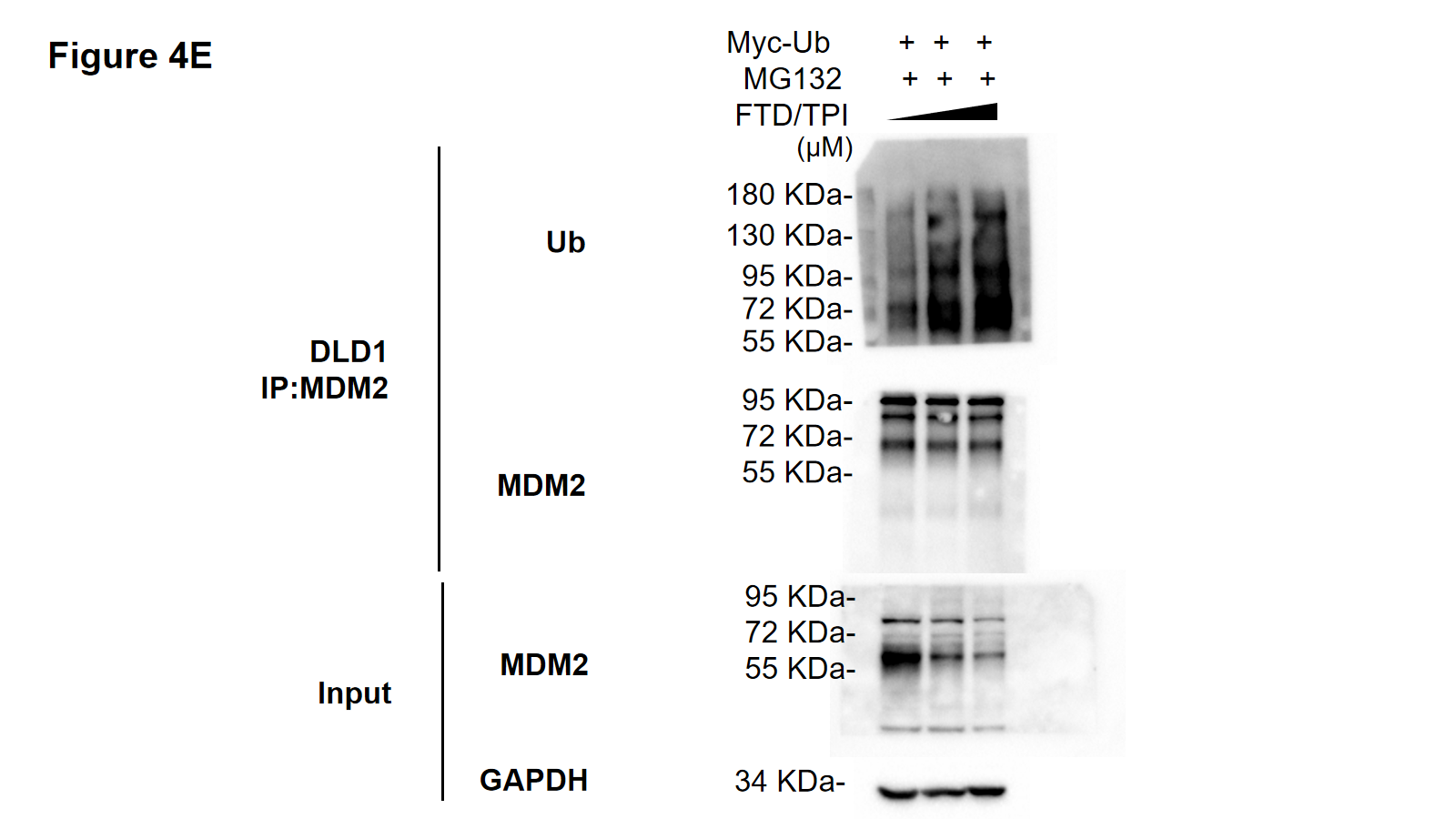

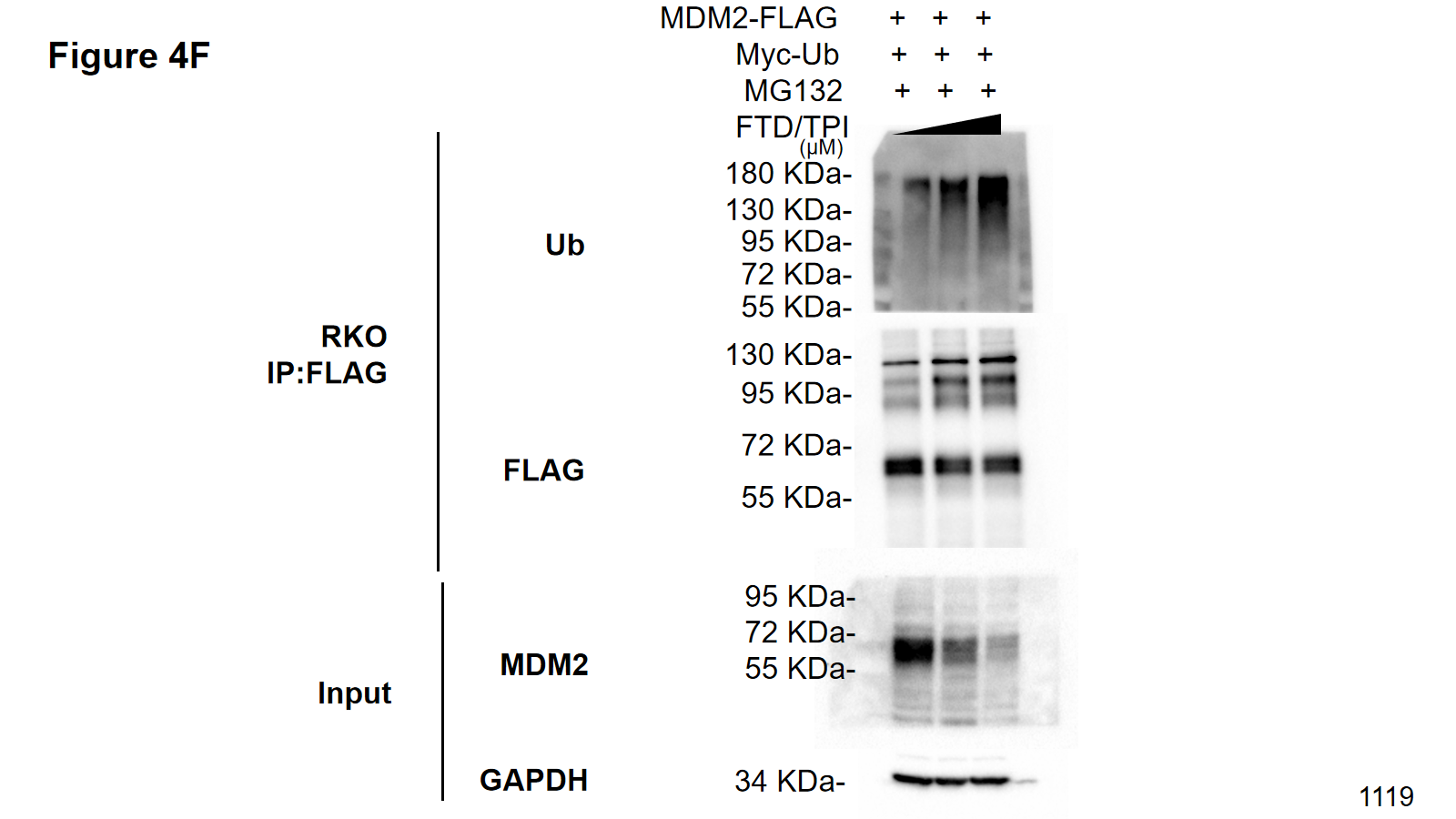

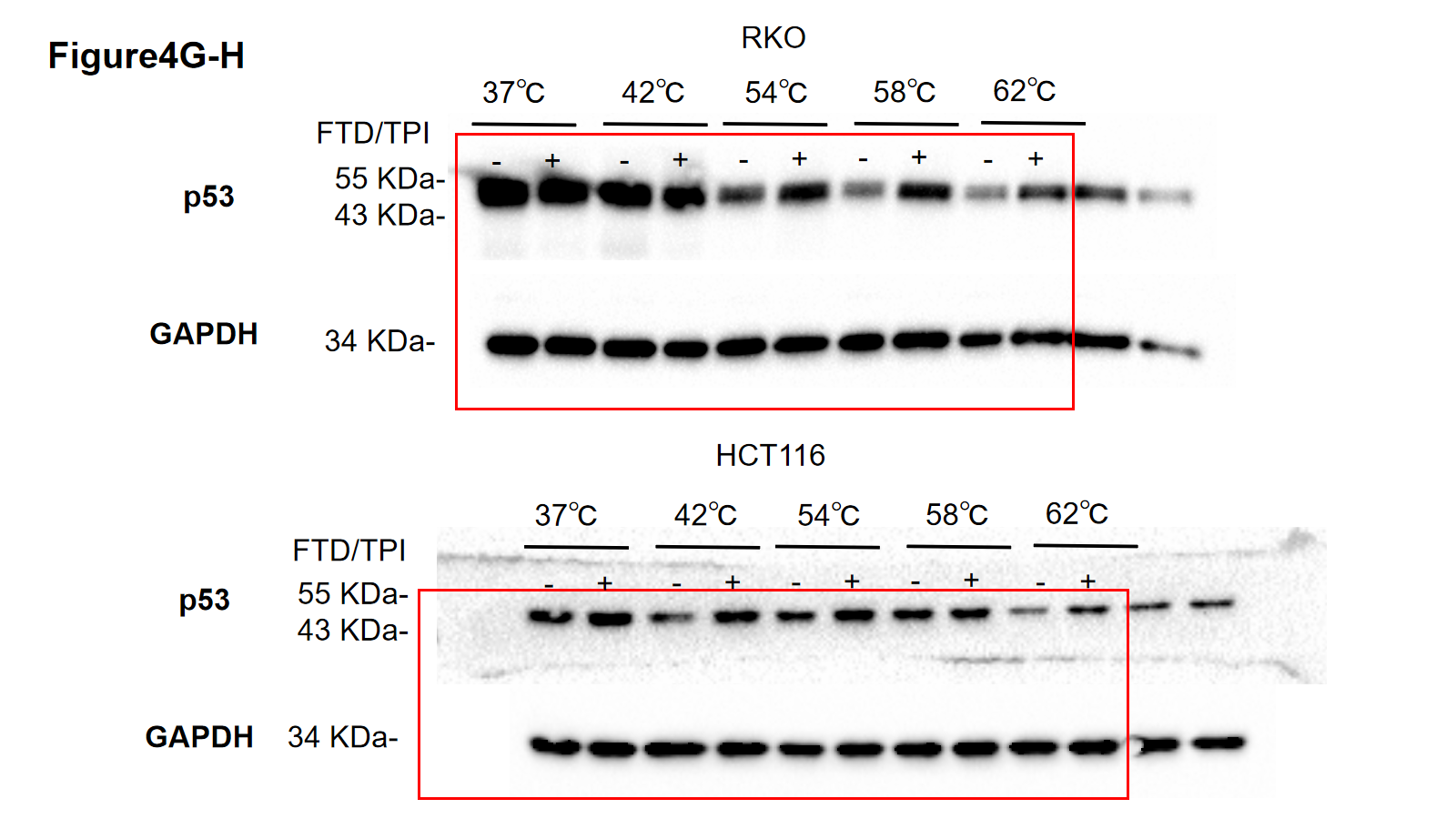

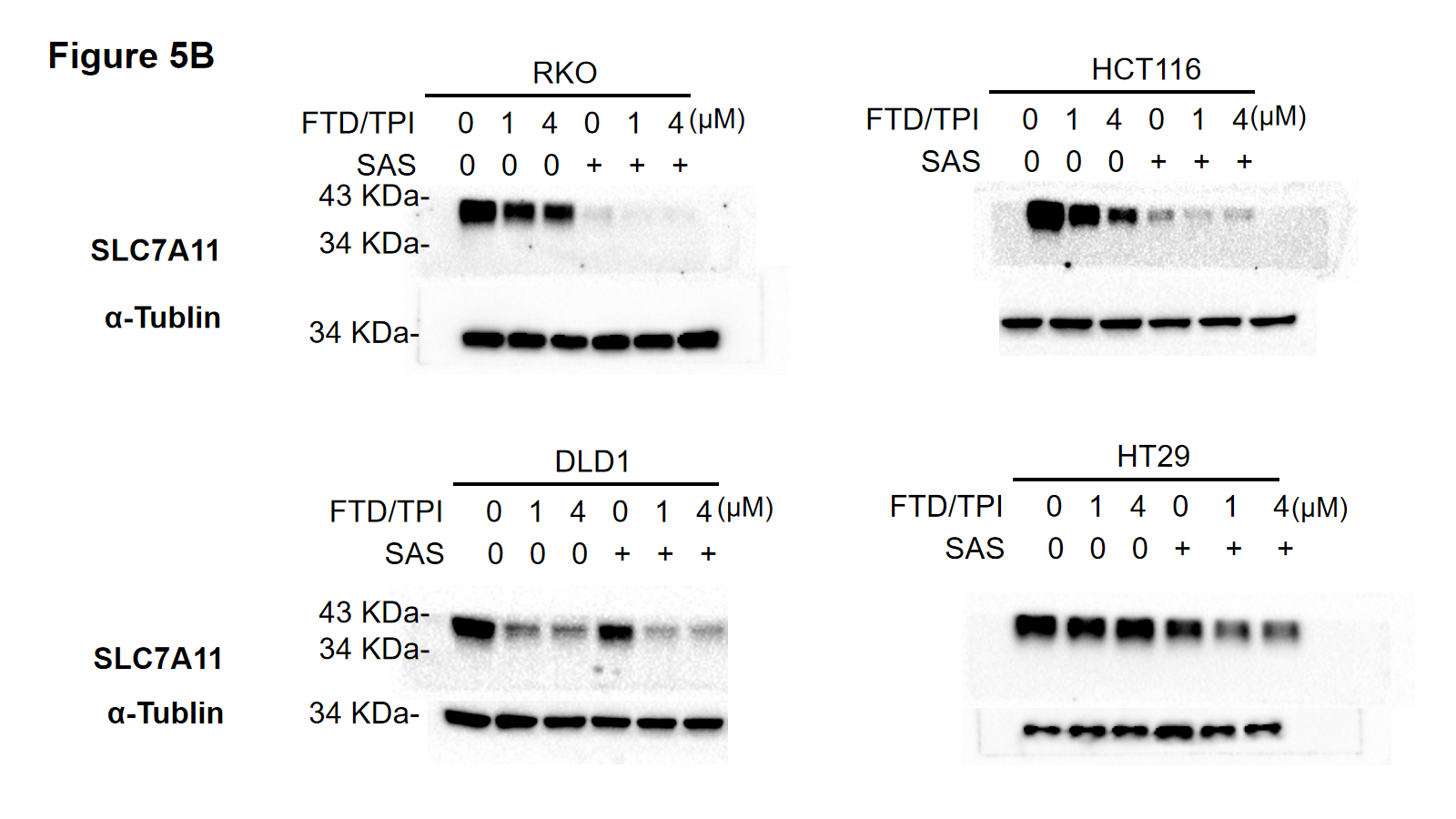

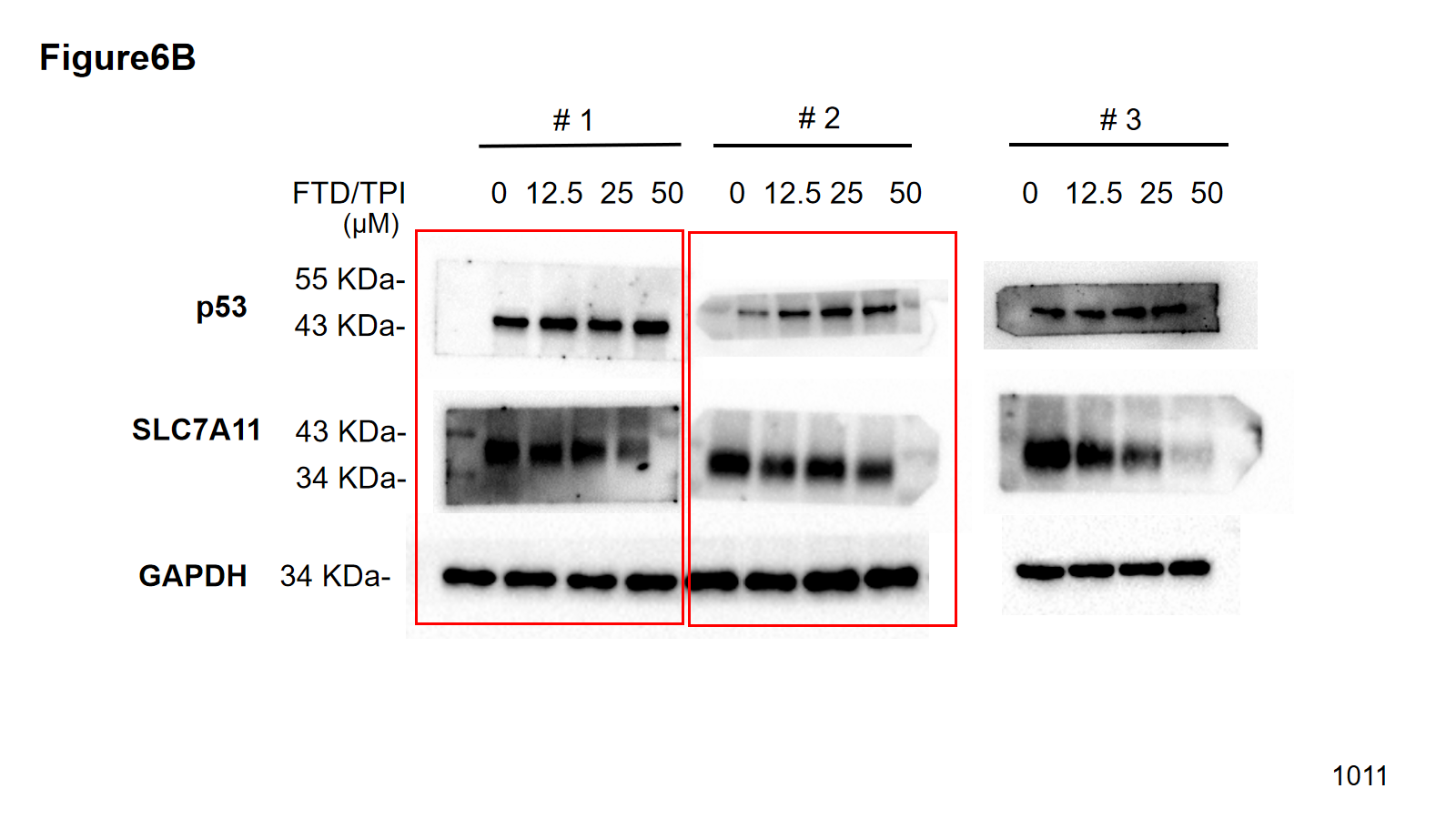

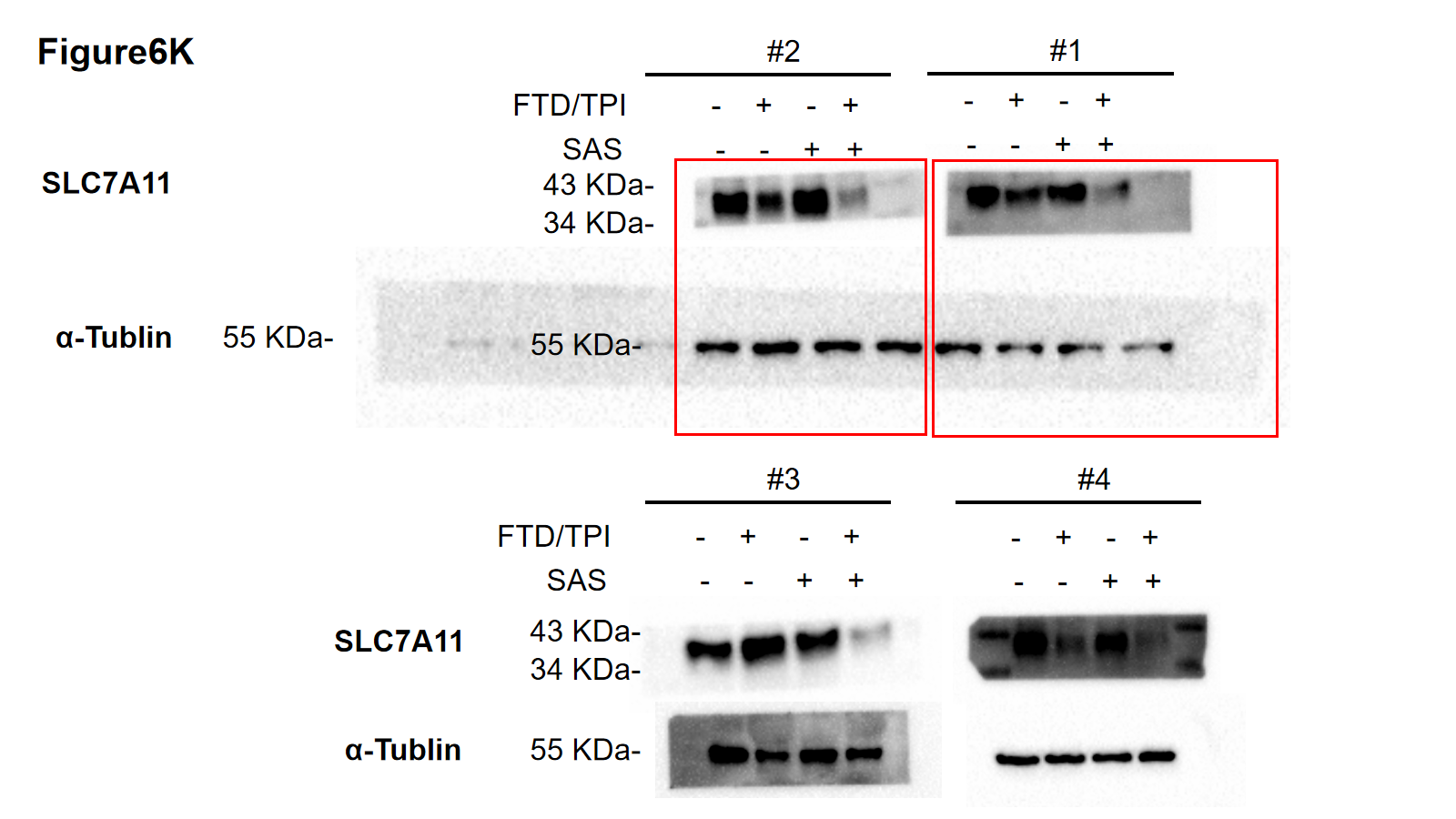

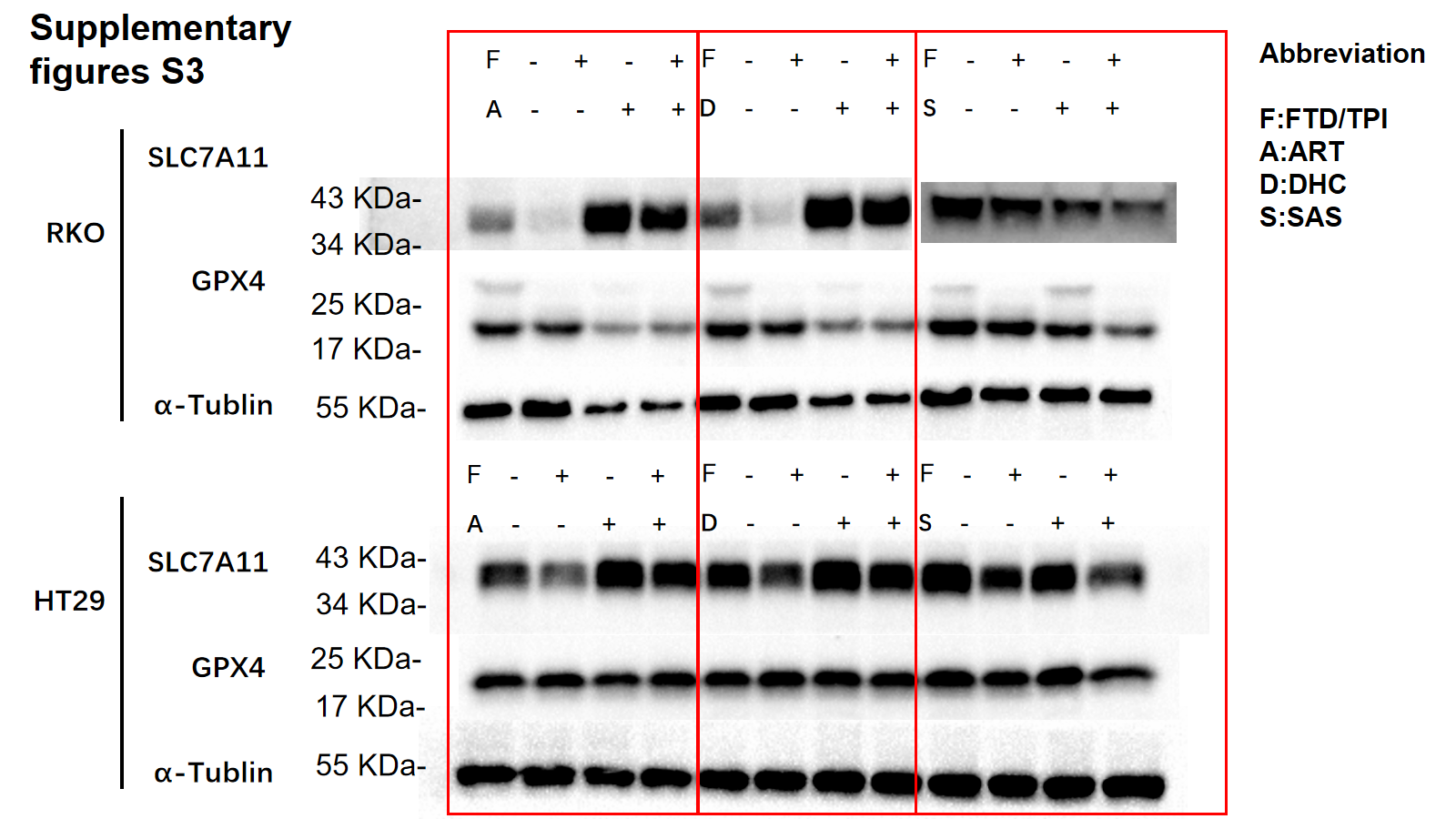

Supplement: Supplementary file 1 — Western Blot of Manuscript [file 41419_2025_7541_MOESM1_ESM.docx]
