# Supplementary material for: Trifluridine/tipiracil induces ferroptosis by targeting p53 via the p53-SLC7A11 axis in colorectal cancer 3D organoids
Source: Cell Death Dis. 2025 Apr 5;16(1):255. doi: 10.1038/s41419-025-07541-z (PMC11972347; doi:10.1038/s41419-025-07541-z)
Supplement: Supplementary file 2 — Supplementary Materials (clean version) [file 41419_2025_7541_MOESM2_ESM.docx]

**Supplementary Materials for**

**Trifluridine/Tipiracil induces ferroptosis by targeting p53 via the p53-SLC7A11 axis in colorectal cancer 3D organoids**

Maosen Huang^1#^, Yancen Wu^1#^, Linyao Cheng^1^, Lihua Fu^1^, Xiaoxia Wei^1^, Haochao Yan^1^, Wene Wei^1,2,4^, Bo Li^5^, Haiming Ru^1,2,3^, Xianwei Mo^1,2,3^, Weizhong Tang^1,2,3^, Zijie Su^1,2,4#^ and Linhai Yan^1,2,3#^

**Corresponding author:**

Linhai Yan, Department of Gastrointestinal Surgery, Guangxi Medical University Cancer Hospital. Nanning, 530021, Guangxi Zhuang Autonomous Region China.

Email: [yanlinhai000@163.com](mailto:yanlinhai000@163.com), Phone: 0086-771-10421

Zijie Su, PhD, Department of Experimental Research, Guangxi Medical University Cancer Hospital. Nanning, 530021, Guangxi Zhuang Autonomous Region China.

Email: [zijiesu@126.com](mailto:zijiesu@126.com), Phone: 0086-771-10421

^#^ These authors contributed equally to this work.

**This file includes:**

**Supplementary table. S1**

**Supplementary figures. S1 to S6**

**Supplementary Materials and Methods.**

**Supplementary table S1**

Table S1 Primer sequences used for real-time PCR amplification.

| **Gene** | **Sense (5’-3’)** | **Anti-sense (5’-3’)** |
| --- | --- | --- |
| TP53 | CAGCACATGACGGAGGTTGT | TCATCCAAATACTCCACACGC |
| GPX4 | ACAAGAACGGCTGCGTGGTGAA | GCCACACACTTGTGGAGCTAGA |
| SLC7A11 | TCTCCAAAGGAGGTTACCTGC | AGACTCCCCTCAGTAAAGTGAC |

**Supplementary figures S1**


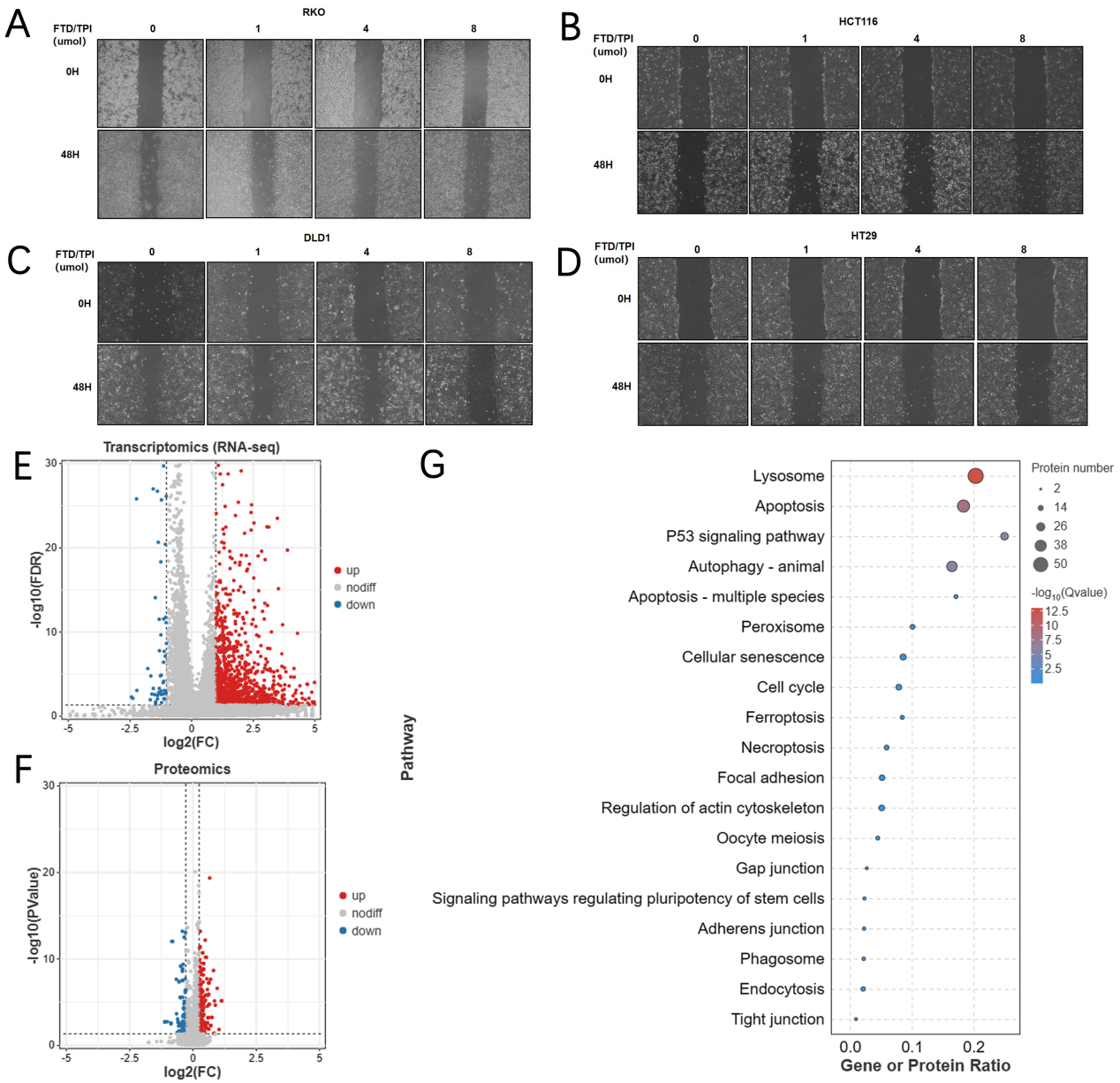


**Fig. S1 Multi-omics correlation analysis of FTD/TPI treatment in colorectal cancer cell lines.** (A-D) Morphological images of scratch experiments on RKO, HCT116, DLD1, and HT29 cells under concentration gradients (0, 1, 4, 8 μmol of FTD/TPI) at 0 and 48 hours. (E) Volcano plot of transcriptomic sequencing data comparing FTD/TPI-treated RKO cells with the control group after 48 hours. Red represents upregulated genes, blue represents downregulated genes. The horizontal axis represents Log2 (fold change) indicating the differential magnitude, and the vertical axis represents -log10 (FDR) or -log10 (p-value) indicating the statistical significance. (F) Volcano plot of proteomic sequencing data comparing FTD/TPI-treated RKO cells with the control group after 48 hours. (G) Bubble plot of metabolically relevant KEGG pathways obtained from the cross-analysis of transcriptomic and proteomic data.

**Supplementary figures S2**


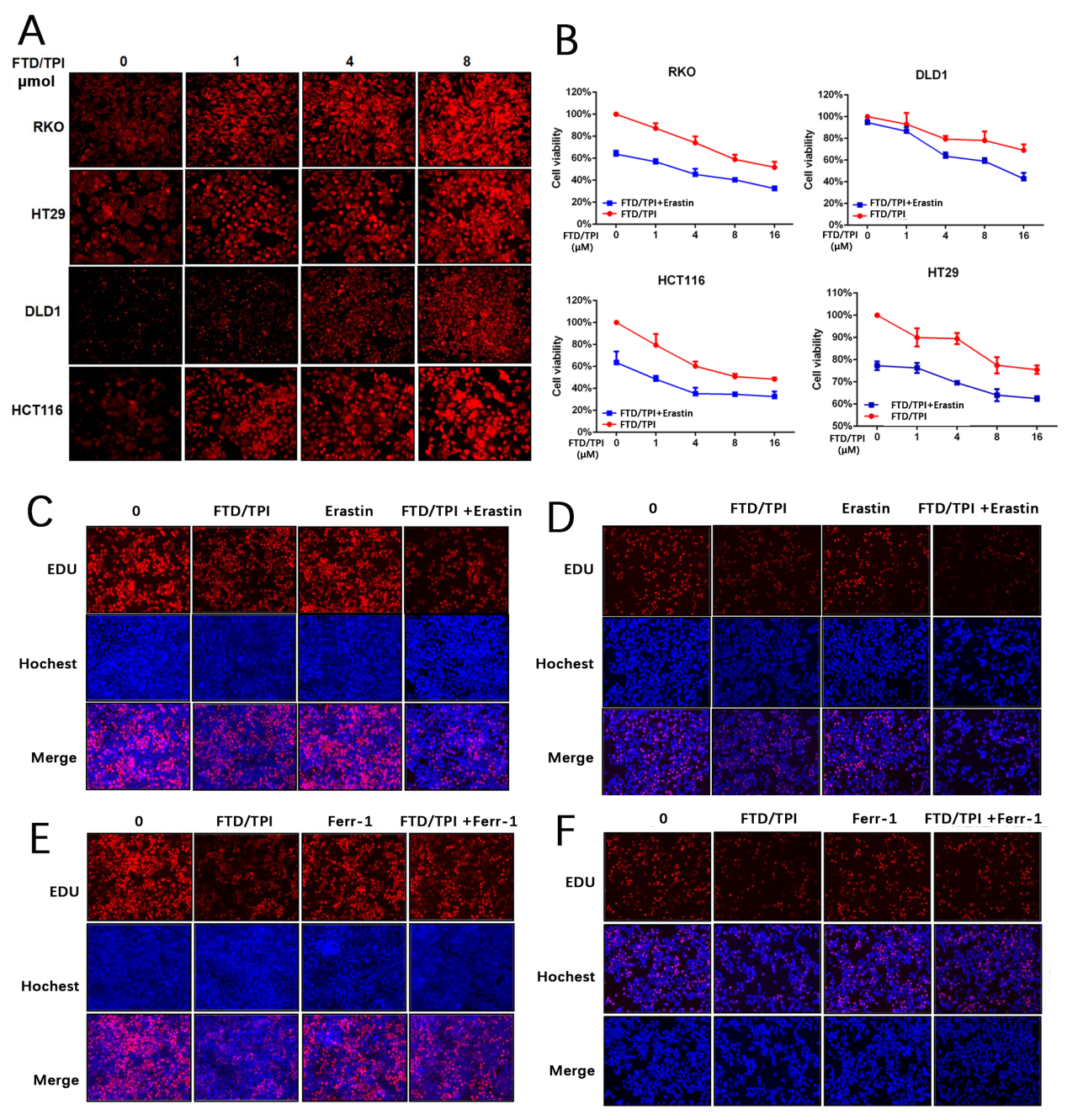


**Fig. S2 Inhibition of cell proliferation by FTD/TPI in the presence of ferroptosis activators and inhibitors.** (A) Fluorescence spectra of iron ion deposition in RKO, HCT116, DLD1, and HT29 cells cultured at concentrations of 0, 1, 4, and 8 μmol for 48 hours under FTD/TPI conditions. (B) Cell viability assay of RKO, HCT116, DLD1, and HT29 cell lines treated with gradient concentrations of FTD/TPI (0, 1, 4, 8, 16 μM) and Erastin (1 μM) for 48 hours. (C-D) EDU staining to evaluate proliferation in RKO and HT29 cell lines treated with FTD/TPI (0, 1, 4, 8 μM) and Erastin (1 μM) for 72 hours. The first row represents red fluorescence for EDU, the second row represents blue fluorescence for Hochest, and the third row represents merged fluorescence. Scale bar = 100 μm. (E-F) EDU staining to evaluate proliferation in RKO and HT29 cell lines treated with FTD/TPI (0, 1, 4, 8 μM) and Ferrostatin-1 (2 μM) for 72 hours. **p* < 0.05, ***p* ≤ 0.01, ****p* < 0.001, *****p* ≤ 0.0001, indicating statistical significance.

**Supplementary figures S3**


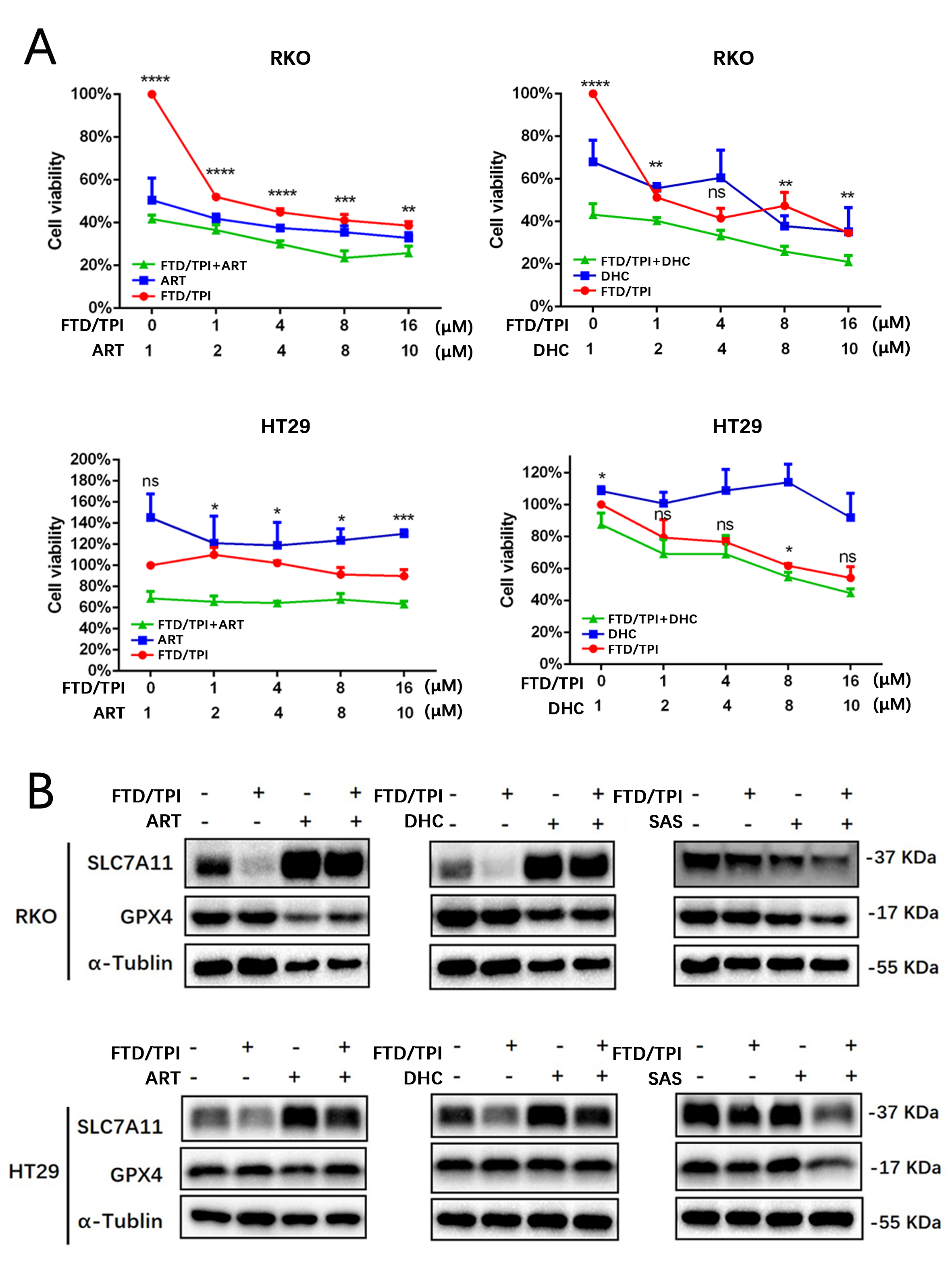


**Fig. S3 Combination drug screening of FTD/TPI with ferroptosis inducers.** (A) Cell viability assay (MTT) of FTD/TPI (0, 1, 4, 8, 16 μM) in combination with Artemisinin (ART. 0, 1, 2, 4, 8, 10 μM) and Dihydroartemisinin (DHC. 0, 1, 2, 4, 8, 10 μM) in RKO and HT29 cells after 48 hours. (B) Western blot analysis of FTD/TPI (4 μM) in combination with ART (4 μM) and DHC (4 μM) after 48 hours, measuring the protein expression of SLC7A11 and GPX4. **p* < 0.05, ***p* ≤ 0.01, ****p* < 0.001, *****p* ≤ 0.0001, indicating statistical significance.

**Supplementary figures S4**

**
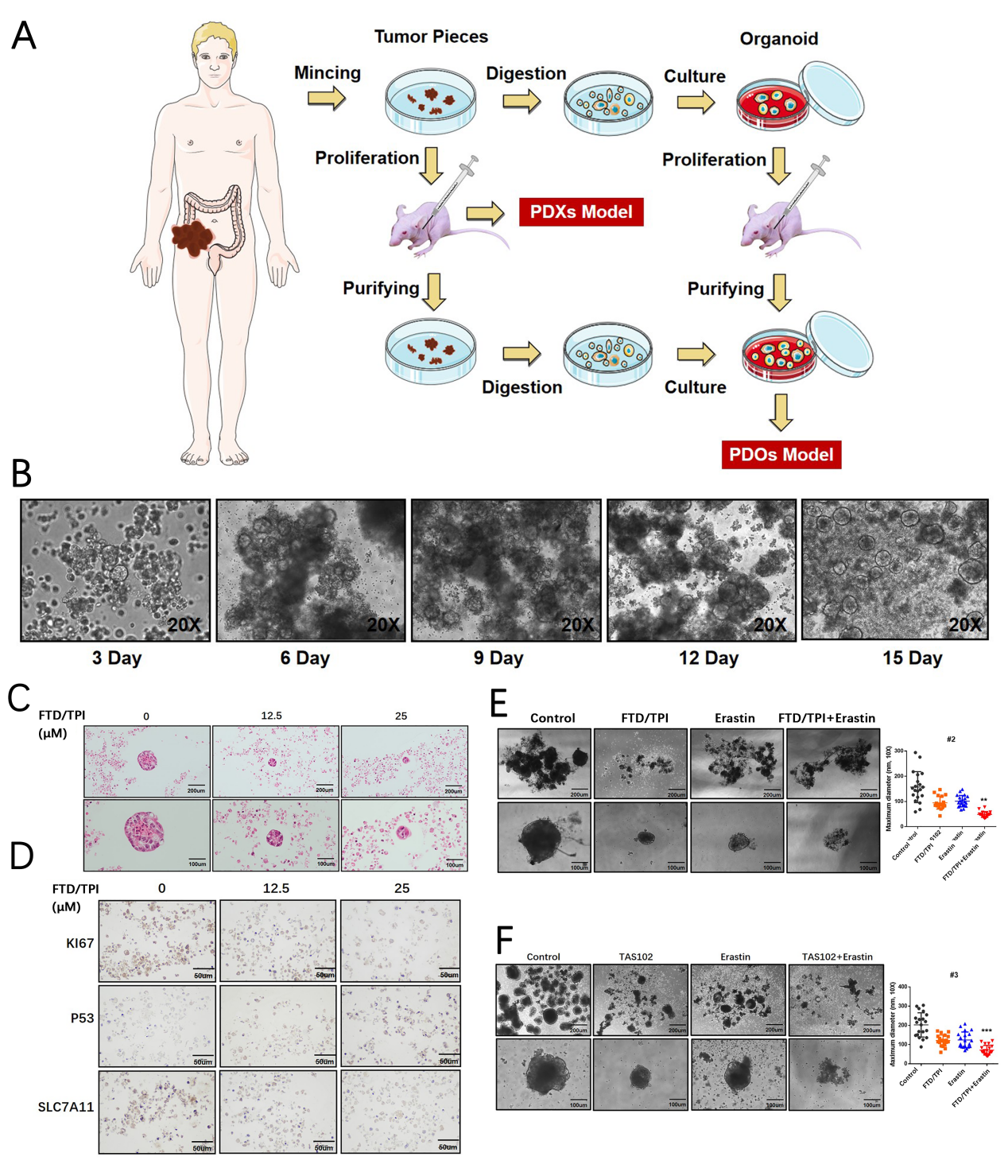
**

**Fig. S4** (A)Process diagram of organoid culture system. (B) Morphological diagram of organoid growth under the microscope. (C) Hematoxylin and eosin (HE) staining of pathological sections from CRC organoids treated with FTD/TPI (0, 12.5, 25 μM) for 7 days. (D) Immunohistochemical analysis of protein expression levels of KI67, P53, and SLC7A11 in CRC organoids after treatment with FTD/TPI (0, 12.5, 25 μM) for 7 days. (E) Microscopic images of CRC organoids from three patients were captured after treatment with 12.5 μM FTD/TPI and 1 μM Erastin for 7 days to observe and quantify organoid size. Scale bar=200 μm, magnification at 4X and 20X. Data are presented as representative images or mean ± SD from at least three independent replicates. Statistical analysis was performed using a two-tailed paired t-test. **p* < 0.05, ***p* ≤ 0.01, ****p* < 0.001, *****p* ≤ 0.0001, indicating statistical significance.

**Supplementary figures S5**

**
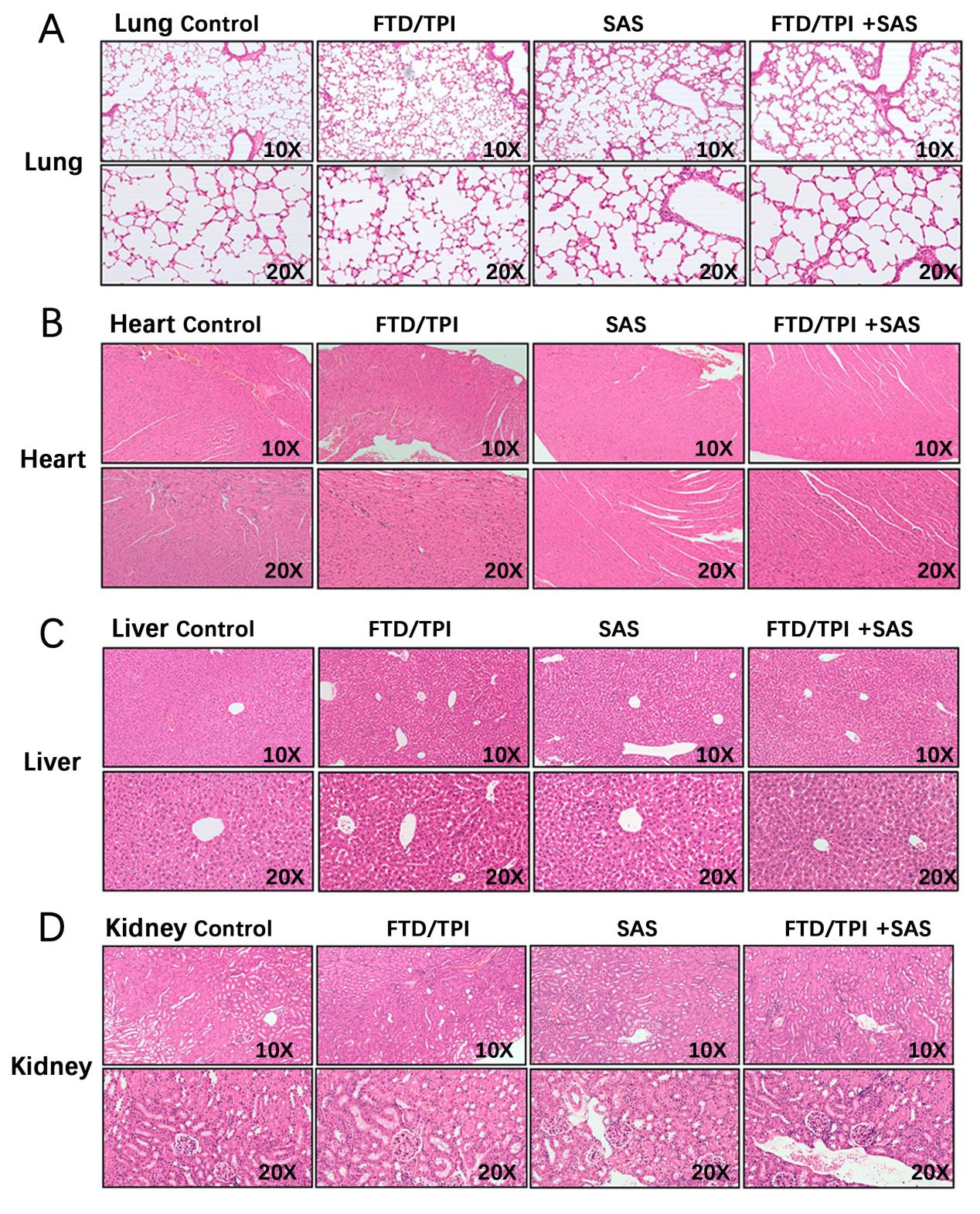
**

**Fig. S5 Evaluation of mouse tolerance to combination drug treatment.** (A-D) HE-stained histopathological sections of the heart, liver, lung, and kidney from PDX model mice in the control group, FTD/TPI group, sulfasalazine group, and combination drug group after euthanasia.

**Supplementary figures S6**


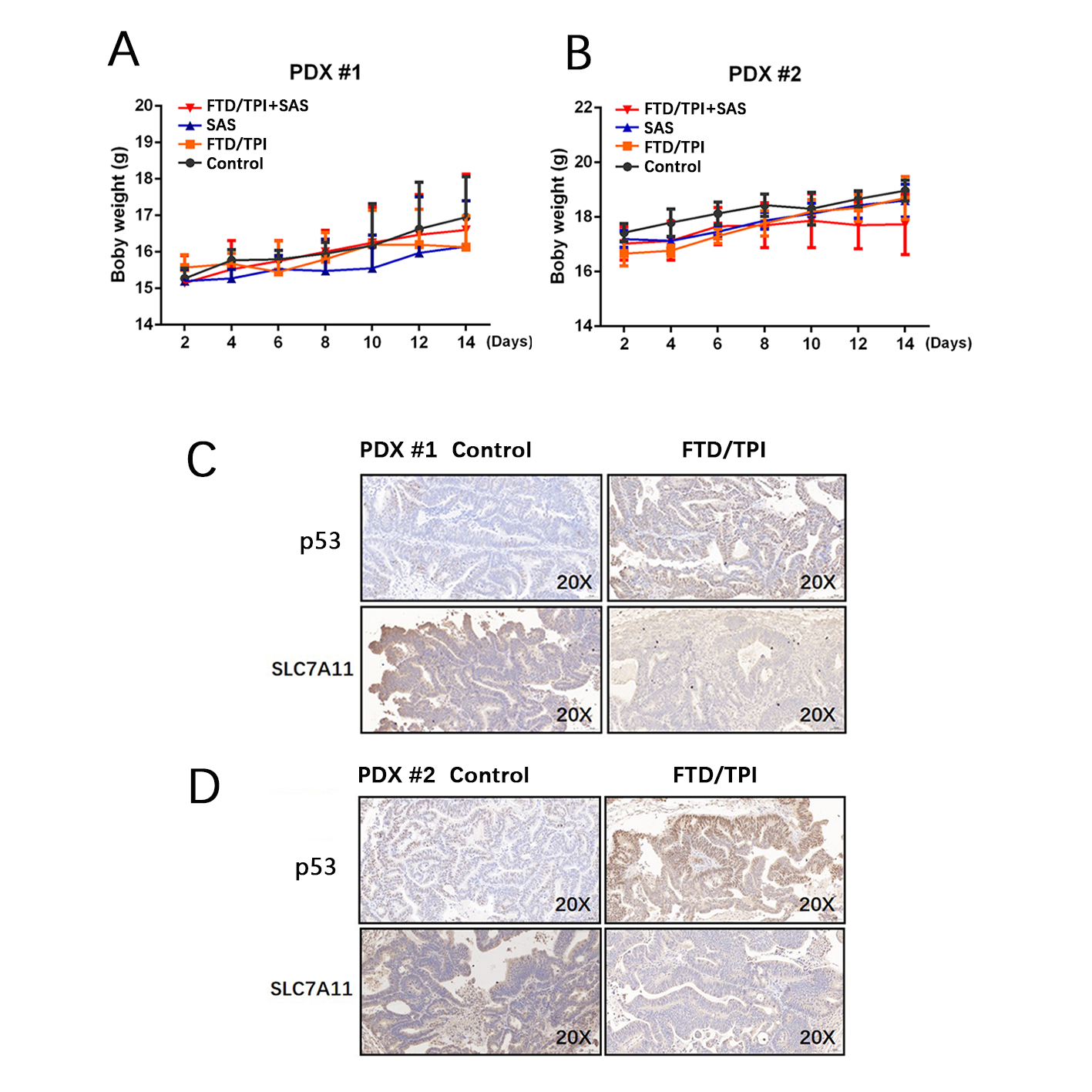


**Fig. S6 FTD/TPI in combination with ferroptosis inducer sulfasalazine in PDX models.** (A-B) Average daily changes in mouse weight for each group in two PDX models. (C-D) Immunohistochemical observation of p53 and SLC7A11 protein expression in the control group and FTD/TPI group after tumor collection in two PDX models. Data are presented as representative images or mean ± SD from at least three independent replicates. Statistical analysis was performed using a two-tailed paired t-test. **p* < 0.05, ***p* ≤ 0.01, ****p* < 0.001, *****p* ≤ 0.0001, indicating statistical significance.

**Supplementary Materials and Methods**

**Cell lines**

The human CRC cell lines RKO and HT29 and the human embryonic kidney cell line HEK293T were cultured in DMEM medium (Gibco), HCT116 and DLD1 cells in RPMI-1640 medium (Gibco). Cells were supplemented with 10% fetal bovine serum (Gibco) and 10000 U/mL Penicillin-Streptomycin (Gibco), and incubated at 37 ℃ with 5% CO2. All cells were obtained from the American Type Culture Collection (ATCC, Manassas, VA, USA) and tested for mycoplasma.

**Chemical regents, antibodies and plasmids**

TAS102 (FTD/TPI), MG132, cycloheximide, and Bafilomycin A1 were obtained from Selleck ((Houston, TX, USA). Ferrostatin-1, Necrosulfonamide and Z-VAD-FMK were purchased from Targetmol (Wellesley Hills, MA, USA). Erastin, sulfasalazine, artemisinin, and dihydroartemisinin were obtained from MedChemExpress (Monmouth Junction, NJ, USA). The following primary antibodies were used: p53 (Santa Cruz, sc-126), ACSL4 (Santa Cruz, sc-365230), MDM2 (Cell Signaling Technology, #86934), SLC7A11 (Cell Signaling Technology, #12691), Ubiquitin (Proteintech, 10201- 2-AP), GPX4 (Abcam, ab125066), GAPDH (Proteintech, 60004-1-Ig), α-Tubulin (Proteintech, 66031-1-Ig). The plasmid of human MDM2 constructed in pCMV-MCS-3×Flag vector was a gift from Mailgene (Mailgene, Beijing, China, MH03043).

**Cellular thermal shift assay（CETSA）**

In order to determine the targeting effect of FTD/TPI on p53, a cellular thermal shift assay was performed as described [31-32]. RKO and HCT116 cells were placed in a 15 cm Petri dish and treated with specific concentration of FTD/TPI or DMSO for 1 hour. Cells were collected and washed with PBS once, and then suspended in PBS with protease inhibitors (Monmouth Junction, NJ, USA), maintaining the same dose of FTD/TPI or DMSO as the initial treatment. The cell suspension was distributed in five 1.5 mL PCR tubes at different designated temperatures. The samples were heated for 2 minutes in a metal bath heater at different specified temperatures. Then the tubes were removed and immediately incubate at room temperature for 3 minutes. Three repeated freeze-thaw lysis of cells in liquid nitrogen were performed. Cell lysate was collected by centrifuging at 20000 g for 20 minutes at 4 ℃. The sample of cell lysate was boiled in loading buffer at 95 ℃ for 5 minutes for western blotting analysis, and the dissolution curve of p53 protein was prepared.

**Molecular docking**

To evaluate the binding affinity and interaction mode between the candidate TAS102 and its target p53, we used Autodock Vina 1.2.2, a computer software for protein-ligand docking. The molecular structure of FTD (PubChem CID：6256) was obtained from the PubChem Compound database. The 3D coordinates of the protein p53 (PDB: 8DC8) were downloaded from the Protein Data Bank. The protein and ligand files were prepared by converting all protein and molecule files to the PDBQT format, removing all water molecules, and adding polar hydrogen atoms. The grid box was centered to cover the structural domain of each protein and accommodate free molecular motion. The docking pocket was set as a square pocket with dimensions of 30 Å × 30 Å × 30 Å and a grid spacing of 0.05 nm. Molecular docking studies were performed using Autodock Vina 1.2.2 for model visualization.

**Transmission electron microscopy**

RKO and HCT116 cells were placed in a 6 cm Petri dish and treated with a specific concentration of FTD/TPI or DMSO. Cells were pre embedded with agar, fixed and dehydrated at room temperature: The sample was infiltrated and embedded, polymerized, and ultrathin sectioned for staining. The copper mesh was placed in a 2% uranium acetate saturated alcohol solution and a 2.6% lead citrate solution to avoid carbon dioxide and light for 8 minutes, respectively. Clean and dry overnight at room temperature. Photograph were captured with a transmission electron microscopy (hitachi, HT7800) under 2500, 7000 and 15000 folds of microscope. The mitochondrial volume and mitochondrial membrane density of the cells were managed with ImageJ software.

**FerroOrange assay**

Colorectal cancer cells were inoculated in 12 well plate (1x10^5^), and after adding gradient-concentration FTD/TPI and culturing for 48 hours, they were washed three times in HBSS and stained with 1 μmol/L Ferroorange (DOJINGO) in HBSS for 30 minutes in a 37℃ incubator with 5% CO2, and imaged immediately. Images were then collected using a fluorescence microscope. Five representative areas were captured under each condition at the same exposure time.

**ROS and MDA assay**

The cells were cultured in a 12 well plate (1x10^5^). After a certain period of drug treatment, add CM-H2DCFDA dye (Invitrogen) and BODIPY™ 581/591 C11 dye (Invitrogen) dissolved in DMSO. Enable CM-H2DCFDA, BODIPY™ Mix 581/591 C11 to achieve a final concentration of 0.5 μmol/L and 5 μmol/L dye solution reacts and incubates in a dark environment. Finally, flow cytometry was used to read FITC fluorescence channels, and flowjo analysis was performed to derive the results. The values obtained from three independent replicates of each cell line under each condition were used to generate a histogram as quantitative data. CM-H2DCFDA and BODIPY™ 581/591 C11 should be configured for the determination of ROS and MDA in Organoid of colorectal cancer with a final concentration of 1 μmol/L and 10 μmol/L React with M's dye solution, and finally take fluorescence microscope photos to observe the fluorescence intensity of each group, including green sunlight (CM-H2DCFDA) and red fluorescence (BODIPY™ 581/591 C11), blue fluorescence (Hochester), Merge fluorescence images, and use ImageJ software to calculate fluorescence intensity.

**GSH assay**

The cells were cultured in a 12 well plate (1x10^5^). After a certain period of drug treatment, collect the cell supernatant. Add the GSH reaction solution (Nanjing Jiancheng Bioengineering Institute) to the cell supernatant for reaction, and measure the absorbance values of each well at 405nm using a multifunctional microplate reader (Molecular Devices). And calculate the sample protein concentration using the BCA kit (Nanjing Jiancheng Bioengineering Institute). Calculate the GSH content by combining the measured absorbance OD value and sample protein concentration.

**Quantitative real-time PCR**

Total RNA was isolated from cells using TRIzol reagent (Invitrogen) and cDNA was generated using reverse transcription kit (TaKaRa). Real time fluorescence quantitative PCR was performed with primers (Supplementary table S1) using SYBR Premix Ex TaqII (Promega) and three duplicate samples were run on a software 7500 instrument. Normalize the threshold period (Ct) of the target gene to the threshold of GAPDH and use 2^- ΔΔ Ct^ calculates the relative expression level of target genes.

**Western blotting**

Use RIPA buffer to lyse proteins in cells, which contains 50 mM Tris HCl, pH 7.4, 150 mM NaCl, 1% Triton X-100, 1% Na deoxycholate, 1 mM EDTA, 0.1% SDS, and add protease inhibitors to obtain proteins through ultrasonic lysis. The protein was then loaded in equal amounts and separated with polyacrylamide gel. Then transfer the protein to the PVDF membrane. After incubating the first and second antibodies, they reacted with ECL solution and detected signals in the Biorad chemidoc MP system.

**CRISPR knock out cell line**

TP53 was knocked out in DLD1 cells by using CRISPR/Cas9 technology. The single-guide RNA targeting sequence GATCCACTCACAGTTTCCAT was cloned into the LentiCRISPRv2 vector to obtain the TP53 KO plasmid. For lentiviral production, HEK293T cells were transfected with a packaging plasmid (psPAX2), an envelope plasmid (pMD2.G), and a TP53 KO plasmid using Lipofectamine 3000 regent according to manufacturer’s instruction. At 48 h after transfection, the supernatants was collected, filtered through a 0.45 mm filter, and centrifuged at 20,000 rpm for 2 h at 4 °C to harvest virus particles. Virus was immediately added to DLD1 cells with 8 μg/mL polybrene. After infection for 4 days, cells were selected for stable expression in the presence of 3 μg/mL puromycin for one week. The puromycin resistant stable clones were pooled. The TP53 deficiency was confirmed by Western blotting and quantitative real-time PCR.

**Human tissue samples, organoid culture, and PDX modelling.**

All human tissue samples who had not been received any chemotherapy or radiotherapy prior to surgery were obtained from Guangxi Medical University Cancer Hospital. All patients provided written informed consent to allow any excess tissue to be used for research studies. The study was approved by the ethics committee of Guangxi Medical University Cancer Hospital.

The organoid culture was performed as descried [33]. Briefly, Tissues were minced into small pieces and digested in digestion buffer (200 U/ml type IV collagenase, 125 μg/ml type II dispase, and 50 U/ml deoxyribonucleic I in PBS) at 37 °C for 1 hour. Cells were cultured with growth medium (500 ng/mL R-Spondin1, 100 ng/mL Noggin, 40 ng/mL EGF, 20 ng/mL FGF-basic, 10 μM Y-27632, 10 mM Nicotinamide in advanced DMEM/F12 ) at 37 °C, 5% CO2. The organoid growth medium was refreshed every two to three days.

Establishment of PDX was performed as previous described [34]. Necrotic areas and adipose tissue were removed following surgery. Around 1 mm3 of tumor fragments were subcutaneously (s.c.) implanted into the right flank of male NPG mice using a trocar. Successfully engrafted tumor models were then passaged and banked after three passages in mice. After successful expansion of the F3 generations of two patient-derived colorectal tumor specimens in NPG mice, the F3 PDX fragments were cut in pieces in about 1 mm3 and implanted s.c. into the right flank of male NPG mice. Tumor growth was measured every 3 days. When the tumors reached about 50 mm3, mice were randomly divided into four groups and intraperitoneal (i.p.) injected with the vehicle (PBS), TAS102 (150mg/kg), SAS (250mg/kg), or TAS102 (150mg/kg) combine with SAS (250mg/kg) every day for 5 days as the first round. Two days later, the mice were managed for a second round treatment the same as before for another 5 days. Mice weight and tumor volume were recorded every 2 days. Tumor sizes were measured with a caliper and tumor volumes were calculated using the formula: 0.5 ×length × width2. Mice were sacrificed in two days after the second round treatment, and tumors were collected and photographed.

**Immunohistochemistry**

Tissue and cell precipitation were fixed with 4% Paraformaldehyde for 48 hours, and then embedded in paraffin to make 4 μ Slice m, perform HE staining and immunohistochemistry staining, take photos under a microscope, and collect immunohistochemical images of each group. Immunohistochemical primary antibodies include Ki67 (Servicebio, GB121141-100), p53 (Servicebio, GB12626-100), and SLC7A11 (Affinity, #DF12509).

**Transcriptomics**

After treating RKO cells with TAS102 for 48 hours, total RNA was extracted using the Trizol method, and the quality of the extracted RNA was checked using agarose gel electrophoresis. Subsequently, a transcriptome library was constructed by mixing the first-strand reaction buffer with random primers, isolating mRNA, fragmenting and adding primers, synthesizing the first and second strands of cDNA, preparing cDNA library fragment ends, followed by adapter ligation, purification of ligation reaction mixture, and finally PCR library enrichment and purification. The obtained data were subjected to differential enrichment analysis, with Log2FC fold change used as the criterion for differential gene selection, followed by GO functional and KEGG pathway enrichment analysis.

**Proteomics**

After treating RKO cells with TAS102 for 48 hours, total cellular proteins were extracted and the protein concentration was determined using the BCA assay kit. SDS-PAGE electrophoresis and Coomassie brilliant blue staining were performed for 1 hour, followed by destaining with destaining solution. The protein bands should be clear, and differences compared to the control group can be observed in the treated samples. Subsequently, the proteins were subjected to reduction, alkylation, acetone precipitation, washing, and enzymatic digestion. The digested samples were taken out, and appropriate desalting columns were used for desalting and peptide quantification according to experimental needs. Finally, spectral databases and DIA data acquisition and analysis (nano-HPLC-MS/MS analysis and data acquisition) were established. Protein annotation was then performed, including GO and KEGG annotation.

**Statistical methods**

The data in this study were analyzed using SPSS software (SPSS 23.0, Chicago, Illinois, USA) and GraphPad Prism Version 9.0 software (La Jolla, CA, USA). Continuous numerical variables in both groups were expressed as mean ± standard error of the mean (SEM). Statistical significance for group comparisons was determined using t-tests or analysis of variance (ANOVA), and a p-value < 0.05 was considered statistically significant. All in vitro experimental results were obtained from at least three independent biological replicates.
